# Significance evaluation in factor graphs

**DOI:** 10.1186/s12859-017-1614-z

**Published:** 2017-03-31

**Authors:** Tobias Madsen, Asger Hobolth, Jens Ledet Jensen, Jakob Skou Pedersen

**Affiliations:** 1 Department of Molecular Medicine, Aarhus University, Palle Juul-Jensens Boulevard 99, Aarhus, Denmark; 2 Bioinformatics Research Center, Aarhus University, C.F. Møllers Allé 8, Aarhus, Denmark; 3 Department of Mathematics, Aarhus University, Ny Munkegade 118, Aarhus, Denmark

**Keywords:** Significance evaluation, Factor graph, Saddlepoint approximation, Importance sampling

## Abstract

**Background:**

Factor graphs provide a flexible and general framework for specifying probability distributions. They can capture a range of popular and recent models for analysis of both genomics data as well as data from other scientific fields. Owing to the ever larger data sets encountered in genomics and the multiple-testing issues accompanying them, accurate significance evaluation is of great importance. We here address the problem of evaluating statistical significance of observations from factor graph models.

**Results:**

Two novel numerical approximations for evaluation of statistical significance are presented. First a method using importance sampling. Second a saddlepoint approximation based method. We develop algorithms to efficiently compute the approximations and compare them to naive sampling and the normal approximation. The individual merits of the methods are analysed both from a theoretical viewpoint and with simulations. A guideline for choosing between the normal approximation, saddle-point approximation and importance sampling is also provided. Finally, the applicability of the methods is demonstrated with examples from cancer genomics, motif-analysis and phylogenetics.

**Conclusions:**

The applicability of saddlepoint approximation and importance sampling is demonstrated on known models in the factor graph framework. Using the two methods we can substantially improve computational cost without compromising accuracy. This contribution allows analyses of large datasets in the general factor graph framework.

**Electronic supplementary material:**

The online version of this article (doi:10.1186/s12859-017-1614-z) contains supplementary material, which is available to authorized users.

## Background

Factor graphs are a graphical model formalism, able to capture both Bayesian networks and Markov networks, i.e. directed and undirected graphical models [1]. Graphical models enjoy widespread use in genomics, in diverse areas such as genetics, integrative genomics and comparative genomics [2–4]. A range of well-known bioinformatical models, such as position weighted matrices, hidden Markov models, hierarchical models and phylogenetic models can all be cast into the factor graph formalism. Therefore the overall return from efficient algorithms and methods operating on factor graphs is high.

Signals in data are often associated with large deviation from a null (noise) model. The amount of deviation is quantified with a score, such as the odds-ratio, i.e. the ratio between the probability of an observation under a foreground model to the probability under a null model. The odds-ratio is a popular choice of score, but in statistical practice other scores can be preffered, either because they are more robust, easier to compute, easier to interpret or simply because there is no explicit foreground model. Irrespectively of the chosen score function, an important question is the statistical significance of the score, i.e. what is the probability that a score as high or higher is generated from the null model.

In the present paper we consider the problem of evaluating statistical significance of rare events defined over factor graphs. A problem which is generally NP-hard even in the special case where all variables are independent of one another [5, 6]. Accordingly, it is important to formulate numerical approximations instead of exact methods. We have developed two approximation methods, one is based on importance sampling, the other on a saddlepoint approximation. Both methods rely on novel algorithms for their efficient evaluation. The merits of the individual methods are assessed both theoretically and in a simulation study.

The applicability of the methods is demonstrated with four models used in different areas of bioinformatics. First, we consider the Poisson binomial distribution. This model has a number of applications, among others in cancer driver detection, where it is used to find regions of the genome that contain a surprisingly high number of somatic mutations [7]. Second, the ubiquitous position weight matrix model for motif description is discussed. The literature on PWM models also contains proofs that the problem of evaluating the significance of a motif match score defined by a PWM under a genomic background represented by a first-order Markov model is NP-hard [5, 6]. This also shows that the more general class of problems is NP-hard. The third model is higher-order Markov chains. Again a Markov chain is an extremely versatile model with applications both inside and outside of bioinformatics. Here we focus on a recent use in modelling sequence motifs, where parameters in a higher-order Markov chain are learned in a regularized fashion [8]. Finally we look at phylogenetic models and models of nucleotide substitution. Phylogenetic models are interesting in their own right but also serve to illustrate the use on models with more complex dependency structures. We use the framework to evaluate if a position in an alignment column shows evidence of evolutionary conservation. Though simplified this is conceptually similar to the widely used phyloP-score, measuring evolutionary conservation and acceleration [9].

For many probabilistic models it can be computationally expensive if not intractable to evaluate the statistical significance of an observation. Even for the models where an efficient computational scheme exists it is often time-consuming to derive and implement. With the genericity of the factor graph formalism, we believe that the methods proposed here, will aid the analysis of data using a wide range of models. We have implemented the importance sampling and saddlepoint approximation methods in a freely available R-package called dgRaph and provide code for the examples discussed in the paper. For efficiency the core algorithms are implemented in C++ using the Rcpp R-package [10]. dgRaph also contains methods for training factor graph models using the EM-algorithm, this however is not a focus in the current paper, where we will treat models and parameters as given.

Despite the fact that the saddlepoint approximation was conceived as far back as 1954 [11], it has only seen sporadic use in genomics [12, 13]. We find that there are ample opportunities to apply saddlepoint approximation in genomics, but its intimidating appearance may have prevented more widespread application. By supplying an R-package we hope to reduce the barriers towards the use of saddlepoint approximation.

In applications of importance sampling, the proposal distribution is often picked based on experience, calibration or experimentation. By pointing out similarities between saddlepoint approximation and importance sampling and tying it up to existing litterature, we can advise the choice of proposal distribution in importance sampling on factor graphs. Applying this more principled approach could lead to the discovery of more effecient importance sampling schemes for particular problems.

## Methods

### Problem statement

**Fig. 1:**
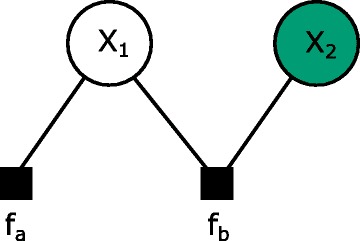
A factor graph with two variables. The probability function is *p*(*x*_1_,*x*_2_)∝*f*_*a*_(*x*_1_)*f*_*b*_(*x*_1_,*x*_2_). It is customary to shade observed variables and leave latent variables unshaded. To calculate the marginal probability of the observed variables, we need to sum out the latent variables. The sum-product algorithm does that efficiently taking advantage of the conditional independence structure of the graph

Throughout the paper we will work with factor graphs [14, 15]. Importantly, both directed (Bayesian networks) and undirected (Markov random fields) graphical models can be cast into the factor graph formalism ([16], ch. 8). A factor graph is a bipartite graph consisting of variable nodes, $\mathcal {X}$, and factor nodes $\mathcal {A}$ (Fig. 1). There can only be edges between variables and factors. To every factor node, *a*, we associate a potential, *f*_*a*_(·), which is a non-negative function of the neighbouring variables, *x*_*a*_. The factor graph induces a probability measure over the variables

1 $$ P(x) \propto \prod\limits_{a \in \mathcal{A}}f_{a}\left(x_{a}\right).  $$

If $\sum _{\mathcal {X}}\prod _{a \in \mathcal {A}}f_{a}\left (x_{a}\right) = 1$ we will say that the factor graph is normalised and the proportionality in (1) can be replaced with equality. The sum-product algorithm, the main algorithm for calculating likelihoods and marginals, operate on graphs free of undirected cycles and with finite state spaces [15]. We will therefore limit ourselves to cycle-free graphs with finite state spaces. As continuous distributions can be discretized and thus this does not present a major limitation.

We define a score of an observation, *x*, as:

2 $$ S(x) = \sum\limits_{a\in\mathcal{A}} g_{a}\left(x_{a}\right),  $$

where $\left \{g_{a}(\cdot)\right \}_{a\in \mathcal {A}}$ is a collection of functions. Given a null model, $\left (\mathcal {X}, \mathcal {A}, \left (f_{a}\right)_{a\in \mathcal {A}}\right)$, we are interested in determining how often extreme scores occur, that is we will address the problem of evaluating significance, *P*(*S*(*x*)>*t*).

It has been shown that even the simpler subclass of this problem where the variables are independent, i.e. each variable node form a connected component together with its neighbouring factor nodes, is NP-hard (see section *Markov Chains*), yet an exact solution can be obtained with a method known as convolution ([17], Ch. 7). The convolution approach may be generalized to the depedence scenarios, factor graphs can represent, but not without significant additional bookkeeping, rendering the method intractable even in problems of modest size. In this light we investigate a number of approximation methods namely naive sampling, importance sampling, normal approximation and saddlepoint approximation.

In many real world problems, for instance in genomics, interesting findings often has significance, *z*=*P*(*S*(*x*)>*t*), in the order of 10^−6^ or smaller. Where an absolute error of e.g. 10^−5^ is more than good enough for a probability in the order of 10^−2^, it is inadequate for a probability in the order of 10^−6^. Generally, if $\hat {z}$ denotes our estimate, to establish the order of magnitude of *z*, we need a small relative error, $\left |z-\hat {z}\right |/{z}$, rather than a small absolute error, $\left | z - \hat {z} \right |$.

### Scores

A number of different scores can be employed, indeed the examples will give an idea of the flexibility that Eq. (2) offers in devising the scoring scheme. Two choices deserve special attention. First, consider the case where we have a null model *P* ^*bg*^(·) and define *S*(*x*)=− log(*P* ^*bg*^(*x*)). With this choice of score a high value is equivalent to a small likelihood indicating an observation that is unlikely under the model. We can write

3 $$ S(x) = -\log(P(x)) = \sum\limits_{a\in\mathcal{A}}-\log\left(\,f_{a}(x_{a})\right)  $$

and it can be seen immediately that *S*(*x*) is indeed of the form (2) with *g*_*a*_(*x*_*a*_)=− log(*f*_*a*_(*x*_*a*_)).

Second, if instead we want to compare a background model to a foreground model, *P* ^*fg*^(·), we can define the score from the ratio of the probabilities in the two models:

4 $$ S(x) = \log\left(\frac{P^{fg}(x)}{P^{bg}(x)}\right) = \sum\limits_{a\in\mathcal{A}}\log\left(\frac{f^{fg}_{a}(x_{a})}{f^{bg}_{a}(x_{a})}\right).  $$

Again this is on the form (2) with

5 $$ g_{a}\left(x_{a}\right)=\log\left(\frac{f^{fg}_{a}\left(x_{a}\right)}{f^{bg}_{a}\left(x_{a}\right)}\right).  $$

### Sampling based methods

In the following we introduce three different approximation methods for significance evaluation. In the end of the section we highlight similarities and differences.

The first method is importance sampling (IS) using the following class of proposal distributions parameterised by *α*:

6 $$ \tilde{P}_{\alpha}(x) \propto P(x)\exp\left(\alpha \sum\limits_{a\in \mathcal{A}}g_{a}\left(x_{a}\right)\right).  $$

As *α* increases the corresponding proposal distributions will generate higher scores more frequently. Note that by taking *α*=0, IS is reduced to naive sampling. With the particular choice of $\left \{g_{a}\right \}_{a \in \mathcal {A}}$ from Eq. (5), the proposal distributions have the form

7 $$ \tilde{P}_{\alpha}(x) \propto P_{bg}(x)^{1-\alpha}P_{fg}(x)^{\alpha}.  $$

Here the parameter *α* gradually skews the proposal distribution from the background distribution (*α*=0) to the foreground distribution (*α*=1) and beyond.

Due to the factorisation properties, the proposal distributions generally have a particularly simple form

8 $$ \begin{aligned}
\tilde{P}_{\alpha}(x) & \propto P(x)\exp\left(\alpha \sum\limits_{a\in \mathcal{A}}g_{a}\left(x_{a}\right)\right) \\ & = \prod_{\mathcal{A}} f_{a}\left(x_{a}\right) \exp\left(\alpha g_{a}\left(x_{a}\right)\right) \\ & = \prod_{\mathcal{A}} \tilde{f}_{a,\alpha}(x_{a}),
\end{aligned} $$

where $\tilde {f}_{a,\alpha } = f_{a}(x_{a}) \exp \left (\alpha g_{a}(x_{a})\right)$. This is again an (unnormalised) factor graph model with the same structure. The marginal distribution for each variable and for each set of variables neighbouring the same factor node can be found with the sum-product algorithm. Using a method reminiscent of the forward sampling method used for Bayesian networks ([1], p. 488-489), we can generate samples, *x*_1_,*x*_2_,…,*x*_*N*_, from the proposal distribution (8) (Additional file 1: Figure S1). The weight of each sample is $w_{i} = w(x_{i}) = {P(x_{i})}/{\tilde {P}_{\alpha }(x_{i})}$ and the score is *s*_*i*_=*S*(*x*_*i*_). The IS estimate of the significance is then,

$$P(S > t) \approx \frac 1N\sum_{i=1}^{N}w_{i}\mathbb{I}\left(s_{i} > t\right). $$

As with sampling in general the variance of the estimate is $\mathcal {O}(1/N) $, yet the choice of *α* is critical to the performance of IS in practice. One natural choice of *α* is such that the mean score under the importance sampling distribution is equal to the score threshold, *t*, i.e. ${\mathbb {E}_{\alpha }\left [ S \right ] = t}$.

Using proposal distributions of the form (7) has been explored previously in sequence analysis; the same idea is applied to hidden Boltzmann models, a generalization of hidden Markov models, in [18]. This theory enables computation of significance statistics over sequences of arbitrary length, whereas we generalize to arbitrary structures. We will see later that this particular class of proposal distributions is in fact an example of *exponential tilting* ([19], pp. 129-131), an idea tightly linked to the method of saddlepoint approximation that we will explore next. In [18] it is recommended to pick *α* using calibration requiring sampling with multiple different values of *α*’s. Below we provide a method for choosing an *α*, that in many cases has the property of logarithmic efficiency (see *Efficiency*), and can computed efficiently.

### Analytical approximations

We now derive two analytical approximations. First the conceptually simpler normal approximation and second the saddlepoint approximation.

Consider a random variable *S* with density function *f*_*S*_(*s*) and define the moment generating function(mgf), $\varphi (\theta) = \mathbb {E}\left [e^{\theta S} \right ]$, and the cumulant generating function(cgf), *κ*(*θ*)= log*φ*(*θ*). The exponential family generated by *S* is defined by

9 $$  f(s;\theta) = \exp(\theta s - \kappa(\theta))f_{S}(s).  $$

The probability measures in this family are called the *exponentially tilted measures*. The following important identities connect the mean and variance of distributions in this family to the cumulant generating function, see e.g. ([20], p. 6):

10 $$ \mathbb{E}_{\theta}\left[ S\right] =\kappa^{\prime} (\theta) \quad \text{and} \quad \mathbb{V}_{\theta}\left[ S\right] =\kappa^{\prime\prime} (\theta).  $$

In a normal approximation the score distribution is approximated by a normal distribution having the same mean and variance. These quantities can be found using the cumulant generating function:

11 $$ m_{s} = \mathbb{E}\left[S(x)\right] = \kappa^{\prime}(0)  $$

and

12 $$ v_{s} = \mathbb{V}\left[S(x)\right] = \kappa^{\prime\prime}(0).  $$

The tail estimate is then:

13 $$ P(S > t) \approx 1 - \Phi\left(\frac{t - m_{s}}{v_{s}}\right)  $$

where *Φ* is the distribution function of the standard normal distribution. The normal approximations has generally quite poor performance in the tail of the distribution as we will show later.

Saddlepoint approximation (SA) is another analytical approximation that has better performance in the tails ([20], ch. 4; [21]). SA is typically used for independent variables or in weak dependence scenarios [22], but we have developed algorithms that allow their evaluation on general factor graphs. Along with introducing SA, that might be unfamiliar to many readers, we will also indicate where these algorithms are used.

SA proceeds by choosing the parameter, *θ*=*θ*(*t*), called the *saddle-point*, such that the mean under *f*(*s*;*θ*(*t*)) is *t*, that is

14 $$  \mathbb{E}_{\theta(t)}\left[ S\right] = \kappa^{\prime}(\theta(t)) = t.  $$

We want to evaluate the tail probability

15 $$  \begin{array}{ll} P(S > t) &= \int^{\infty}_{t}f_{S}(s) ds \\ & = \int^{\infty}_{t} \exp\Big(-\theta(t)s+\kappa(\theta(t))\Big)f(s;\theta(t)) ds. \end{array}  $$

Now approximate *f*(*s*;*θ*(*t*)) with a normal distribution having the same mean, *t*=*κ* ^′^(*θ*(*t*)), and variance, *v*≡*κ* ^′′^(*θ*(*t*)). Then we have

16 $$\begin{array}{*{20}l} P(S > t) &\approx \int^{\infty}_{t} \exp(-\theta(t)s+\kappa(\theta(t)))\\ &\quad\times\frac{1}{\sqrt{2\pi v}}\exp(-\frac{1}{2v}(s-t)^{2}) ds \\ &= \varphi(\theta(t)) \int^{\infty}_{t} \frac{1}{\sqrt{2\pi v}}\exp\left(-\frac{(s-t+\theta(t)v)^{2}}{2v} \right.\\ &\quad+\left. \frac{1}{2}\theta(t)^{2}v-\theta(t)t\right) ds \\ &= \varphi(\theta(t))\exp(-t\theta(t))\exp\left(\frac{\theta(t)^{2}v}{2}\right)\\ &\quad\times\left[ 1-\Phi(\theta(t)\sqrt{v})\right].  \end{array} $$

In order to obtain the saddlepoint approximation we need to solve (14) and compute *κ* ^′′^(*θ*(*t*)). It turns out that both *κ* ^′^ and *κ* ^′′^ can be calculated exactly with extensions of the standard message passing algorithm (Additional file 1: Figure S9). We solve (14) numerically using Newton-Raphson and then proceed to calculate *κ* ^′′^(*θ*(*t*)).

### Importance sampling vs. saddlepoint approximation

Importance sampling and saddlepoint approximation are more similar than they appear at a first glance. Let us look again at (9), *f*(*s*,0) is the density function of *s*(*x*) with *x*∼*P*, similarly *f*(*s*,*θ*) is the density function of *s*(*x*) with *x* being distributed according to

17 $$ \begin{array}{ll} f(x;\theta) &= \exp(\theta s(x) - \kappa(\theta))P_{bg}(x) \\ &= \varphi(\theta)^{-1}\exp\left(\theta \sum\limits_{a\in \mathcal{A}}g_{a}(x_{a}) \right)P(x). \\ \end{array}  $$

We see that we recover (6) and that importance sampling and saddlepoint approximation are essentially just two strategies for evaluating (15): Either sampling *f*(*s*,*θ*) indirectly through *f*(*x*,*θ*) or approximating *f*(*s*,*θ*) by a normal distribution. The above analysis also suggests that a good choice of *α* for importance sampling around *t* is using the saddlepoint *κ* ^′^(*α*)≈*t*. We will call importance sampling using this strategy for choosing *α* *saddlepoint guided importance sampling* (SG-IS).

## Results

### Poission-binomial

As a first example we discuss the Poisson-binomial distribution. The Poisson-binomial distribution arises as the number of succeses in *N* independent but not neccesarily identically distributed Bernoulli trials. Let *p*_1_,…,*p*_*N*_ be a set of probabilities and $\{Y_{n}\}_{n=1}^{N}$ be independent with *Y*_*n*_∼Bernoulli(*p*_*n*_). Then ${S = \sum _{n=1}^{N}Y_{n}}$ is Poisson-binomial distributed. In the case where *p*_*n*_=*p* the Poisson-binomial reduces to the regular binomial distribution.

The model has seen widespread use in a variety of fields, including genomics, forensics, psychometrics and ecology [7, 23–25]. As an example Melton et al. [7] considers regional somatic mutation status in cancer samples. A logistic regression model is used to determine the mutation rate at each loci for each sample. They then identify cancer-drivers by testing if a given genomic region has a surprisingly high number of mutated samples.

**Fig. 2:**
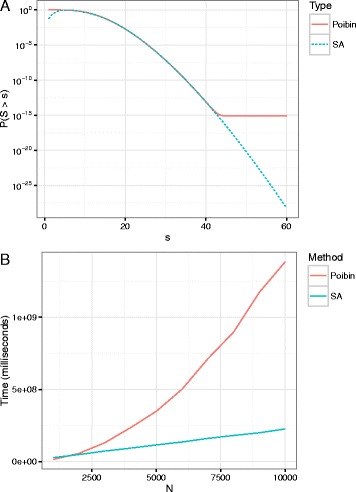
**a** The tail of a Poisson-binomial distribution where *p*_*i*_ is drawn independently from a Beta(1,100) and *N*=1000. The saddlepoint approximation tracks the exact distribution perfectly. The Poisson-binomial as implemented in poibin R-package. **b** A comparison of the computation time for the two algorithms. The DFT-CF method has quadratic run-time complexity whereas the Saddlepoint method has linear run-time complexity

We compute the tail of a Poisson-binomial using SA and using a fast Fourier transform based method (DFT-CF) [26] as implemented in the R-package poibin (Fig. 2 a). In the simple case with *p*_*i*_=*p* we also compare with the exact binomial probabilities (Additional file 1: Figure S10). All comparisons are qualitatively alike: The saddlepoint and DFT-CF methods give identical results for most part of the tail. The saddlepoint approximation is not suited for calculating large (not significant) *p*-values (>0.1). On the other hand the DFT-CF method experiences numerical underflow for small *p*-values (<10^−13^). Large *p*-values are typically not of interest and can usually be computed efficiently by other means.

An additional argument for prefering saddlepoint approximation over DFT-CF is the run-time complexity. Although the DFT-CF uses the fast Fourier transform, the required preprocessing step makes it an $\mathcal {O}(N^{2})$ algorithm. In contrast, the saddlepoint approximation scales linearly with *N*, having complexity $\mathcal {O}(N)$ (Fig. 2 b, Additional file 1: Table S1).

For many applications it is attractive to assign a different score, *s*_*n*_, for each event, *Y*_*n*_, leading to a new score of the form $S = \sum _{n=1}^{N}s_{n}Y_{n}$. Using a different score and thus a different test statistic can be used to increase the statistical power of the test. The DFT-CF does not readily generalize to different scoring schemes whereas this is immediately achieved with SA and SG-IS.

### PWMs

Our next two examples revolve around sequence motifs. We consider analysis of motifs with both the classical *position weighted matrix* model and a more recent Bayesian motif model.

Consider a simplistic DNA model, where DNA is a sequence of letters, *x*_1_⋯*x*_*L*_, from a four-letter alphabet. The *x*_*i*_’s are independent and identically distributed and we let *p*_*j*_=*P*(*x*_*i*_=*j*) for *j*∈{*A*,*C*,*G*,*T*}. A motif is (for our purpose) a fixed length subsequence of DNA that exhibits a specific pattern. This pattern can be described with a probability distribution (*f*_*ji*_)_*j*={*A*,*C*,*G*,*T*}_ at each position *i*∈{1,…,*N*} and is typically represented in a *position weighted matrix* (PWM), which is a 4×*N* matrix, *M*, where *M*(*j*,*i*)=*f*_*ji*_.

If we think of the DNA-model as the background model and the motif as the foreground model, the log score for a subsequence *x*_*m*_⋯*x*_*m*+*N*−1_ of length *N* is simply:

$$S\left(x_{m}\cdots x_{m+N-1}\right) = \sum\limits_{i=0}^{N-1} \log \frac{f_{{ix}_{m+i}}}{p_{x_{m+i}}}. $$

**Fig. 3:**
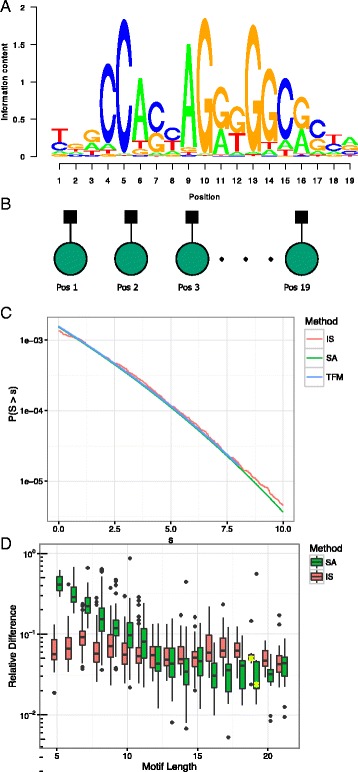
**a** Sequence logo for the CTCF binding motif. The larger the letters, the higher the fold-enrichment compared to the background distribution. **b** The PWM model represented as a factor graph. Note that since the nucleotides are considered independent of one another, no variable nodes are connected. **c** The approximations to the tail obtained from IS, SA and the method from the TFMPvalue package. **d** The relative difference between significance estimates from TFMPvalue and IS and SA respectively for all JASPAR Vertebrate motifs. The differences for the CTCF motif is indicated with *yellow* stars

This is a sum of independent random variables and the motif model can be encoded in a rather simple factor graph, where each variable has its own potential (Fig. 3 b). The significance can be evaluated using discretization and dynamic programming. These computations can be accelerated using heuristics such as branch-and-bound, still the problem remains NP-hard [5, 6].

As an illustration we analyse 1080 motifs from the JASPAR database [27]. Sequence motifs are often represented with so-called sequence logos that show the log2 fold enrichment of a given base relative to the background (Fig. 3 a).

We calculate the significance over a range of scores using both SA and IS and compare with the estimates of the significance obtained from the TFMPvalue software package [5]. Here we show the estimates for the CTCF motif. (Fig. 3 c). As a means for evaluating the difference between the approximations, we compute the relative difference at a number of quantiles and take the average of the numerical value of these. By this measure it can be seen that all three methods agree well: IS showing relative differences in the order of 10% with 1000 samples and without tuning *α*. The relative differences for SA decreases with motif length and is typically less than 10% for motifs longer than 10 basepairs. The motifs where the saddlepoint approximation performs poorly have a strong preference for a single base at each site. For these motifs the score matrix have similar contributions at each site, causing the score distribution to have a discrete nature, not well approximated by SA (Fig. 3 d and Additional file 1: Figure S13).

The Poisson-Binomial and the PWM models can be seen as special cases of a more general class of models with variables taking a discrete set of values. In the supplement we state a theorem giving conditions, where the saddlepoint approximation has uniform relative error $\mathcal {O}(1/N)$ for this class of models (see Additional file 1: Section xiv). We then give sufficient conditions for both the Poisson-Binomial and PWM model. Although this result involves the limiting behaviour of the approximation, it has been demonstrated that the saddlepoint approximation has remarkably small error even for small *N* [21].

TFMPvalue has two modules for *p*-value computation. The first calculates the exact *p*-value and the other rounds the score-matrix to increase computational speed. The exact *p*-value computation module in the TFM software has exponential computational time complexity (Additional file 1: Figure S11) therefore we only compare with the approximate *p*-value calculation from TFM.

The approximate TFMPvalue computation is $\mathcal {O}\left (N^{2}\right)$, but faster in practice due to the branch-and-bound heuristic. Again computing saddlepoint approximation is roughly $\mathcal {O}(N)$. For shorter motifs this does not make any practical difference, but for longer motifs (>20 bp) the difference can be sizeable depending on the exact problem and the desired level of accuracy (Additional file 1: Figure S12). In the next section we will show that the SA and IS methods can be applied to richer motif models, where the convolution methods can not easily be adapted.

### Higher-order Markov chains

First- and higher-order Markov chains is another application domain of SA and SG-IS. In a recent paper by Siebert et al. [8] they argue convincingly for replacing the PWM motif models with higher order Markov chains using a Bayesian prior (BaMMs).

A PWM model assumes that each base in a motif is independent. In contrast Markov chains are able to capture context dependent nucleotide frequencies at the expense of more parameters. Siebert et al. overcome the challenge of training the parameter rich models by employing a Bayesian model, where the prior shrinks the high-order parameters towards their lower-order counterparts for contexts rarely encountered in the training data.

BaMMs outperformed PWMs in discriminating ChIP-seq peak-sequences from simulated background sequences of the same length and tri-mer composition. Including flanking regions widens the gap between BaMMs and PWMs in terms of predictive power. This is possibly explained by two modes of DNA-protein binding specificity; base readout and shape readout. In base readout the protein recognizes the DNA sequence. This form of binding specificity is dominant in the core motif and is reasonably well-captured by PWMs. In shape readout the protein recognizes the shape of the DNA, the DNA shape is in turn determined by motifs showing high neighbour correlation [28].

Due to the large-scale nature of motif-detection accurate *p*-value evaluation is important. As PWMs are Markov chains of order zero, we are again dealing with an NP-hard problem, making it natural to look for approximate methods.

**Fig. 4:**
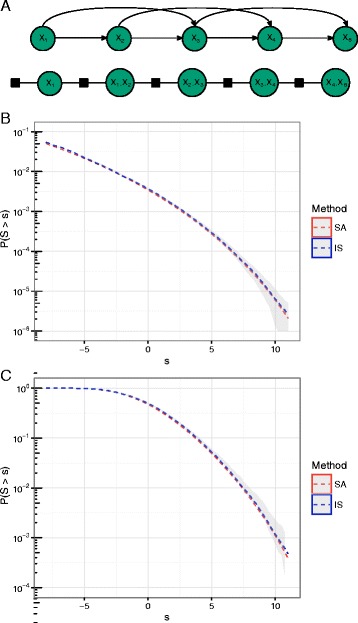
**a** 2nd and higher-order Markov chains contain cycles. But higher-order Markov chains can be viewed as first order Markov chains by compounding variables. We thereby obtain a tree-structured graph. **b** Simulating 10^6^ sequences of the same length as our motif and estimated the significance and corresponding 95% confidence interval shown in shaded *grey*. We compare this with SA and IS using (*α*=0.5) and 10^4^ samples. **c** We simulated 10^4^ sequences of length 200bp and calculated the maximum motif match score of all offsets, the 95% confidence interval is shown in shaded *grey*. To calculate the significance of this maximum, we used the calculation from a single match and employed a Poisson approximation

We obtain a BaMM for the CTCF transcription factor binding motif in MCF-7 cell lines (see Additional file 1: Section x). Second- and higher-order Markov chains contain cycles and are therefore not immediately suited for the framework. However by compounding variables an *n*-th order Markov chain can be represented as a first order Markov chain (Fig. 4 a).

First the significance of the log-odds score of a single match is determined using SA and IS. Second the accuracy of the approximations is verified using deep naive sampling, generating 10^6^ background sequences of the same length as the motif (16bp) with a homogeneous second order Markov model. Comparing the approximation to the estimates obtained from deep naive sampling we the see that they track each other perfectly (Fig. 4 b).

Another classification task of interest is identifying longer sequences containing the motif. We simulate 10^4^ 200 bp long sequences again with a homogenouos second order Markov model. Within a 200 bp long sequence a motif of length *k* can start at 200−*k*+1 positions (offsets). We consider the max of log-odds scores obtained from evaluating a motif match in all offsets. To calculate the significance we use the estimates of significance for a single match and employ a Poisson approximation [29] (see Additional file 1: Section x). The Poisson approximation is typically valid if the sequence we search is sufficiently long and the motif is not of low complexity (i.e. not highly repetitive). Again we observe that the SA and IS method combined with Poisson approximation provides a good approximation of the statistical significance (Fig. 4 c).

### Phylogenetic trees

Our final example is derived from molecular evolution. A phylogenetic tree represents the relationship among species. With each leave representing a species and internal nodes common ancestors.

Evolutionary conservation manifests itself by a slower than normal substitution rate. At the population level, purifying selection maintains phenotypic function by constraining the evolutionary process and effectively eliminates some mutations from being fixed as substitutions. Evolutionary conservation therefore reflects presence of functional constraints. Using a fixed phylogenetic tree and a model for nucleotide substitution we can calculate the expected number of substitutions along the branches of the tree given the present day sequences. We can then evaluate if this number is significantly lower than expected. This is conceptually similar to the widely used phyloP-score, although their method is more sophisticated; modelling and accounting for clade specific mutation rates and indels [9].

**Fig. 5:**
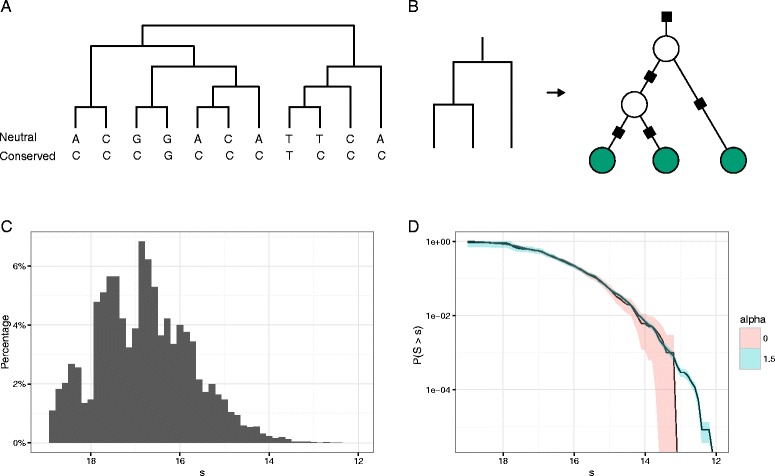
**a** A phylogenetic tree with 11 present sequences. A single alignment column with a high degree of identity across sequences indicate evolutionary conservation. **b** A phylogenetic tree can easily be converted to a factor graph, here shown for a phylogenetic tree with only 3 species. Note that the common ancestors are typically not available for sequencing and their sequences are unknown. The internal variables are therefore unshaded indicating a hidden variable. **c** The distribution of the conditional expectation of the number of substitutions over the whole phylogenetic tree, given the present sequences. The distribution is obtained by simulating 10^5^ times. **d** We use IS to estimate the tail of the distribution by sampling *n*=1,000 scores. Two different *α*-values were used: 0, corresponding to naive sampling and 1.5. Note that naive sampling has much wider confidence bands in the tail compared to importance sampling

We perform our analysis on a phylogenetic tree with 11 leaves, corresponding to present day sequences (Fig. 5 a, for a detailed description of the phylogenetic tree see Additional file 1: Section xii). A phylogenetic tree model can be cast into a factor graph where leaf and each internal nodes are variable nodes and branches are factor nodes (Fig. 5 b). Assuming the Jukes-Cantor substitution model, we can calculate the transition probabilities and the expected number of transitions conditional on the end points of each branch. These are exactly the matrices needed in order to compute the expected number of substitutions conditional on the present-day sequences. Note that we are not limited to the Jukes-Cantor model, these matrices can be computed for any substitution rate matrix [30].

The distribution of the conditional expectation of the number of substitutions is obtained by simulating 10^5^ alignment columns (Fig. 5 c). As we are testing for evolutionary conservation, a low number of expected substitutions is significant, testing for accelertion is however easily done by instead regarding a high number of expected substitutions as significant. While the actual number of substitutions is evidently an integer, the conditional expectation can be any non-negative number. Note also that even for complete sequence identity the expected number of substitutions is non-zero as multiple substitutions at the same site can anull each other. Observing less than 14 expected substitutions is a moderately rare event. But using IS we can get a good handle on these probabilities (Fig. 5 d).

In the present example the chosen score factorizes neatly according to (2), but this would not have been the case had we chosen a likelihood based score. As opposed to the previous examples this example contains latent variables. The log likelihood does not factor into (3). This is not a problem for the IS procedure, where we can still simulate data from the full data distribution and then calculate the likelihood. For the SA method there is no immediate solution.

### Efficiency

In the following two sections we address the question of efficiency; basically establishing and evaluating appropriate measures of the quality of our approximations, both in terms of accuracy and computational cost.

#### Normal and saddlepoint approximations

The error bounds typically given for the normal and saddlepoint approximations, are derived for sums of i.i.d. variables or Markov chains [21, 22]. We review a few of these results. As i.i.d. variables and Markov chains are special cases in our setup, they will inform us on the behaviour we should expect in the general case.

For the normal approximation the Berry-Esseen theorem [31] provides us with an upper bound on the absolute errors. Consider a sequence *X*_1_,*X*_2_,…,*X*_*N*_ of i.i.d. variables having mean *μ* and variance *σ* ^2^. Set $S=\sum _{i=1}^{N} X_{i}$, then

$$\sup\limits_{x \in \mathbb{R}}\left| P(S < t) - \Phi\left(\frac{t-N\mu}{\sigma\sqrt{N}}\right) \right| = \mathcal{O}\left(\frac{1}{\sqrt{N}}\right). $$

However the relative error is not bounded, which in most cases can be ascertained by considering a *t* of order *N*.

On the other hand the saddlepoint approximation has relative error of order $\mathcal {O}(1/N)$ [20]. This bound holds for homogeneuous Markov models and under mild regularity conditions for the Poisson-Binomial and PWM models (see Additional file 1: Section xiv). Opposed to the normal approximation, the saddlepoint approximation will recognize bounded variables in the sense that (14) has no solution if *t* is outside the range of *S*.

To study the behaviour of the saddlepoint approximation in the general case, we conduct a simulation study. We investigated how the complexity of the graph, the degree of independency between the contributions from each factor and the size of the graph affects the quality of the approximation. The graphs were chosen as balanced trees (Additional file 1: Figure S2) and such that the contribution to the sum (2) from each factor had the same marginal distribution. The complexity is adjusted by the degree of the variable nodes in the tree. The degree of independency is measured by the variance ratio, the ratio of the variance of the score and the sum of variances from each factor (i.e. the variance we would have seen if each contribution was independent)

18 $$ VR = \frac{\mathbb{V}\left[ \sum_{a \in \mathcal{A}}S_{a}(X_{a}) \right]}{\sum_{a \in \mathcal{A}}\mathbb{V}\left[ S_{a}(X_{a}) \right]}.  $$

For a more detailed description see Additional file 1: Section ii.

**Fig. 6:**
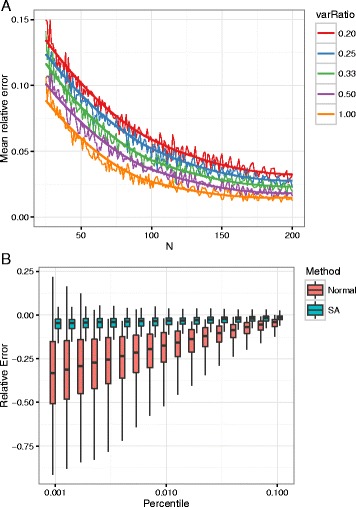
**a** We investigate the quality of the approximation as a function of graph size and conditioned on the degree of independence between variables as measured by the variance ratio (18). Here we found the 1%-quantile in a Markov chain using importance sampling. We then found the saddlepoint approximation of the tail probability in this particular point and plot the relative error as a function of the length of the Markov chain. For details see Additional file 1: Section ii. **b** The relative error measured at different quantiles for both SA and normal approximation, this was done under the same range of conditions as above. We see that while the errors remain stable for the SA they increase for the normal approximation as we move to smaller percentiles

First note that as we go to smaller percentiles the errors in the saddlepoint approximation remains stable, whereas they increase in the normal approximation increases. This parallels the situation for i.i.d. variables (Fig. 6 b, Additional file 1: Figure S3). As expected the relative error decreases with the size of the graph (Fig. 6 a), note however that the errors do not seem to converge to zero. This we believe is explained by the discrete nature of the scores, there exist a correction factor to the saddlepoint approximation in the case the variables take values on a lattice:

19 $$ K(\theta, \alpha) = \frac{\alpha\left|\theta\right|}{1-\exp\left(-\alpha\left|\theta\right|\right)},  $$

where *α* is distance between two consecutive points in the lattice. Generally log-odds scores will not take values on a lattice, still as the correction factor is larger than 1, it suggests that the tail probability is underestimated and more so for large *θ*, this explains the only near convergence to zero. It is further observed that the convergence is slower for more complex graphs, i.e. graphs having many nodes with high degree, and that there appear to be an optimal amount of correlation between the contributions from each factor in the graph (Additional file 1: Figures S4 and S5).

#### Sampling based methods

Both naive sampling and importance sampling gives unbiased estimates. We are therefore concerned with the variance of our estimate and not the bias. Statements about the variance are typically phrased in an asymptotic setup. Let {*P*_*n*_} denote a family of probability distributions, where *P*_*n*_ is derived from a factor graph with *n* factor nodes. Assume also that the contribution of each factor to (2) is identically distributed with mean *μ*. Consider now *z*_*n*_=*P*_*n*_(*S*>*n*(*μ*+*ε*)) and let *Z*_*n*_ be the estimate of a *z*_*n*_ obtained from a single sample. We will say that the class of estimators, $\{Z_{n}\}_{n=1}^{\infty }$, has bounded relative error if

$$\limsup_{n \rightarrow \infty} \frac{\mathbb{V}(Z_{n})}{z_{n}^{2}} < \infty. $$

For technical reasons one often considers the slightly weaker criterion of logarithmic efficiency namely,

$$\limsup\limits_{n \rightarrow \infty} \frac{\mathbb{V}(Z_{n})}{z_{n}^{2-\epsilon}} = 0, \qquad \forall \epsilon > 0. $$

The relationship between naive and importance sampling resembles that between normal and saddlepoint approximation: In the case where the contributions to (2) are also independent, SG-IS has logarithmic efficiency as proven in [19]. For naive sampling the absolute error tends to zero but the relative error tends to infinity. We have strong reasons to believe that if certain regularity conditions regarding the correlation between neighbouring variables are imposed, logarithmic efficiency also holds in the more general case of factor graphs having bounded degree. We are currently working on a proof, this is however beyond the scope of the current work.

### Computational speed

Making a direct comparison of the computational speed of evaluating the saddlepoint and normal approximations on one hand and doing importance sampling on the other is not meaningful, as the accuracy of the importance sampling depends both on the number of samples and the choice of tuning parameter *α*.

Furthermore the three methods have different behaviours when it comes to evaluating a range of points and not only a single point: The additional computing time for importance sampling is negligible as long as the points have roughly similar significance so that we can use a single batch of samples generated with one *α* value. Similarly for the normal approximation the mean and the variance of the score needs being calculated only once, the computational cost of evaluating the normal distribution is again negligible. The saddlepoint approximation does get sligthly faster per evaluation with consecutive evaluation, as the Newton-Raphson procedure can be initiated with the previous saddlepoint, still there is a linear cost in the number of evaluations (Additional file 1: Figures S7 and S8).

All three methods scale linearly with the number of nodes in the graph (Additional file 1: Figure S6). This suggests that we can formulate a rule-of-thumb regarding the number of points we need to evaluate before importance sampling becomes faster than SA. The benchmarks show that one evaluation of the saddlepoint approximation takes about the same time as generating 40 importance samples.

In conclusion saddlepoint approximation is accurate and relatively fast for a single evaluation. If we have to do multiple evaluations importance sampling is preferable. If speed is really the main concern and we need to evaluate a large range of scores, we can use the normal approximation to obtain rough estimates.

## Discussion

The saddlepoint approximation was originally conceived by Daniels as far back as 1954 [11]. Although it has found uses in some areas of biomedical science e.g. survival analysis [32], its intimidating look may have prevented widespread use in genomics, where we believe there is ample opportunity to apply it. The R-package we have developed contains methods for both building and training models, but also for applying the saddlepoint approximation and importance sampling algorithms. Thereby we hope to reduce the barriers towards the use of saddlepoint approximations.

SG-IS was derived by noting similarities between importance sampling and saddlepoint approximation. The literature contains proofs that this importance sampling scheme is in a certain respect the optimal [19]. Taking this more principled way of designing importance sampling distributions is likely to lead to faster convergence to effective importance sampling schemes.

A direction of research that can be further pursued is how to deal with latent variables: As briefly discussed in the context of phylogenetic trees, the log-odds score does not factorize when we have latent variables. It is therefore not possible to calculate the moment generating function and its derivatives efficiently using the algorithms we use here. This prevents the use of the saddlepoint approximation. Importance sampling will however still work, by just using the tilted distribution on the full data distribution.

The methods and algorithms have been phrased in terms of the factor graph formalism throughout the paper. As factor graphs can capture both directed (Bayesian network) and undirected (Markov random field) models the theory applies to both of them. Especially Bayesian networks have gained much popularity as a tool for integrating the vast array of molecular profiling experiments. The general framework of factor graphs is a powerful tool to analyze such data.

## Conclusion

In the current paper we have presented saddlepoint approximation and importance sampling based methods for evaluation of significance in factor graphs. Efficient algorithms were developed for computing the first and second order statistics, required to derive the saddlepoint approximation, making the saddlepoint approximation feasible for large graphs. We provide an adaption of the forward-sampling algorithm tailored to factor graphs, needed for importance-sampling.

We further reviewed the theoretical properties of the two methods. As most results are derived for independent identically distributed variables, a simulation study was performed to confirm that many of the properties still hold in a range of dependence scenarios. Further we compared the computational speed of the methods to give rough guidelines for deciding between the two.

We demonstrated the utility of the methods considering four different bioinformatics applications. The examples were chosen to show that current models can make use of the methods, but also point forward to new applications. First we looked at the Poisson-binomial model, despite or because it is the simplest of the models, it has numerous uses. At the same time it appears that the algorithms used for analysing the Poisson-binomial model is not state of the art. For exact computation, an adaption of the algorithm implemented in the TFMPvalue R-package [5], is likely to outperform the DFT-CF method. We showed that our approximation methods were able to compute the significance to a high accuracy.

Two motif examples were given, both to show that the problem we are solving is NP-hard, but also to provide useful methods to the motif-analysis field. These methods are especially likely to prove valuable for long and complex motifs such as nucleosome binding motifs.

The phylogenetic example was of a more complex nature than the other examples, highlighting the flexibility of the methods, more than trying to compete with existing methods. Yet, it is qualitatively similar to the phyloP-score. With the availability of massive multiple alignments, such as the UCSC 100-way vertebrate alignment and the coming results of the Genome 10K projects [33], there should be ample opportunity to apply these methods.

## Additional file

Additional file 1 Supplementary material. This file contains extended method descriptions and supplementary figures. Additionally there is a vignette accompanying the dgRaph R-package as well as vignettes covering each of the models used in the result section. (PDF 699 kb)

## Abbreviations

BaMM: Bayesian Markov model
DFT-CF: Discrete fourier transform of characteristic function
EM: Expectation maximization
IS: Importance sampling
PWM: Position-weight matrix
SA: Saddlepoint approximation
SG-IS: Saddlepoint guided importance sampling
VR: Variance-ratio
