# Supplementary material for: Significance evaluation in factor graphs
Source: BMC Bioinformatics. 2017 Mar 31;18:199. doi: 10.1186/s12859-017-1614-z (PMC5374669; doi:10.1186/s12859-017-1614-z)
Supplement: Supplementary file 1 — Supplementary material. This file contains extended method descriptions and supplementary figures. Additionally there is a vignette accompanying the dgRaph R-package as well as vignettes covering each of the models used in the result section. (PDF 699 kb) [file 12859_2017_1614_MOESM1_ESM.pdf]

Supplementary Material for Significance Evaluation in Factor Graphs

Madsen, Hobolth, Jensen and Pedersen

Contents

i Sampling from a factor graph . . . 2
ii Simulation study for investigating the Saddlepoint Approximation . . . 4
iii Computational speed . . . 7
iv Algorithm for moment calculation in factor graphs . . . 10
v dgRaph . . . 13
vi Example 1: The Poisson-Binomial distribution . . . 20
vii Poisson-Binomial Vignette . . . 23
viii Example 2: Position Weight Matrices . . . 25
ix PWM Vignette . . . 27
x Example 3: BaMM motifs . . . 29
xi BaMM Vignette . . . 30
xii Example 4: Phylogenetic trees . . . 32
xiii Phylogenetic tree Vignette . . . 33
xiv Asymptotic Results . . . 35
xv Analysis of JASPAR motifs . . . 37

i Sampling from a factor graph

Sampling from a factor graph, can be achieved using a slight modification of the forward sampling algorithm known from Bayesian networks.

1. Calculate variable and factor marginals using the sum-product algorithm.
2. Choose any variable node as a root node and sample from its marginal distribution.
3. Use Depth First Search (DFS) or Breadth First Search (BFS) to traverse the tree.
4. Now there are two cases
   (a) If the node is a variable node we have already sampled it.
   (b) If the node is a factor node we have the marginal distribution of neighbours. We proceed to calculate the conditional distribution given the parent variable and sample all remaining variables from this distribution.

In every step the sampled variables are conditionally independent of the variables already sampled given the parent. This ensures the validity of the procedure.

Example
An example will illustrate the procedure (Fig. S1). We want to sample from a factor graph having 8 variable nodes labelled $A, B, \ldots, H$. Step 1 is a preprocessing step were we obtain the marginal distribution of each variable, and the marginal distribution of each set of variables surrounding the same factor node using the sum-product algorithm. **(i)** In step 2 we sample any variable from its marginal distribution. Here we sample A from $P(A)$. Shading indicates variables that have been sampled. **(ii)** In step 3 start traversing the tree using DFS. In step 4 we first visit the factor node having A, B, C as neighbours. The sum-product algorithm gives us the marginal distribution $P(A, B, C)$. Having already sampled A, we have to condition on A and sample B, C from the conditional distribution $P_{B,C|A}(b, c, a) = \frac{P_{A,B,C}(a,b,c)}{\sum_{i,j} P_{A,B,C}(a,i,j)}$. **(iii)** Next we sample D, again we have to condition on the variables A, B and C that have already been sampled. But D is conditionally independent of A and C given B so $P(D|A, B, C) = P(D|B)$. From the sum-product algorithm we have the marginal $P(B, D)$ and again we can condition on B, $P_{D|B}(d, b) = \frac{P_{B,D}(b,d)}{\sum_i P_{B,D}(b,i)}$. **(iv)** We continue using the same conditional independence argument repeatedly, here sampling from $P(E|D)$. **(v)** Sample from $P(F, G|A)$. **(vi)** Sample from $P(H|C)$.

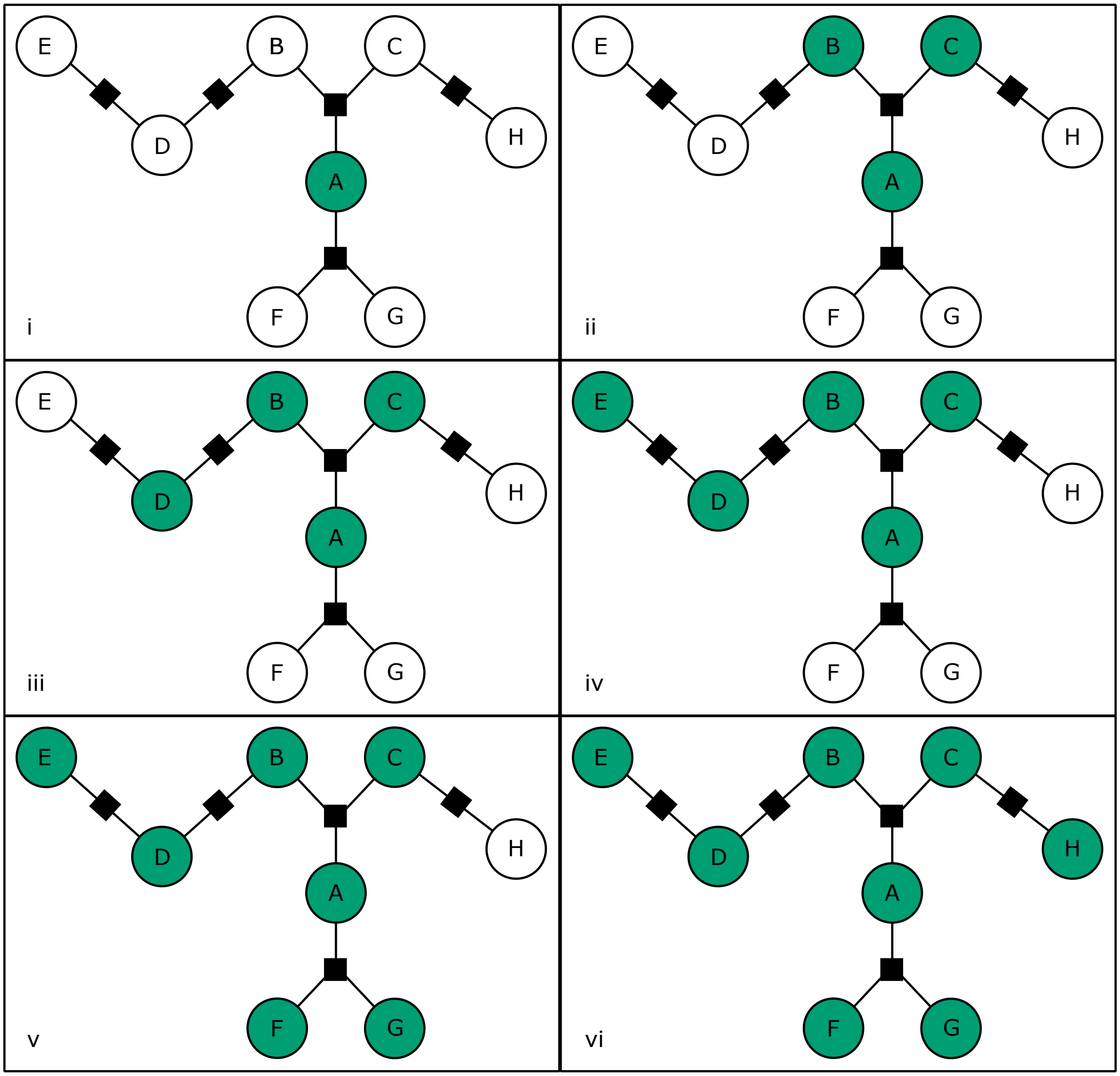

Figure S1: An example showing each stage of the sampling algorithm.

ii Simulation study for investigating the Saddlepoint Approximation

Using a simulation study we evaluate the performance of the saddlepoint approximation under different scenarios. We consider a Markov process on a tree structured graphs with varying degree, Δ and varying number of variable nodes, N. The construction is a follows, label the nodes $0, \ldots, N-1$. Label the root node 0. Connect the i'th node to node $\lfloor \frac{i-1}{\Delta-1} \rfloor$ (Fig. S2). Note that for $\Delta = 2$ we have a Markov chain.

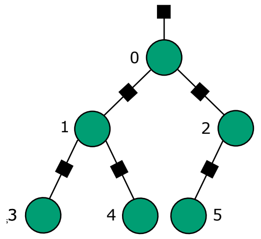

Figure S2: Construction of trees with different complexities here, $\Delta = 3$ and $N = 6$.

Each variable has two states. The transition probability in the background between the two states along any branch is

$$P = \begin{pmatrix} 1-p & p \\ p & 1-p \end{pmatrix}. \tag{S1}$$

The foreground transition probabilities are chosen such that the score associated with each transition is

$$S = K \log \left(\frac{P_{fg}}{P_{bg}} \right) = \begin{pmatrix} \sqrt{2} & 2\sqrt{2} \\ 2 & 4 \end{pmatrix}. \tag{S2}$$

We determine the relative error at 17 quantiles corresponding to tail probabilities in a geometric progression from 10^{-1} to 10^{-3}. Two measures of error were studied, namely the mean and the max of the absolute error over the 17 quantiles.

We use the importance sampling procedure with 10^5 samples to determine the tail probabilities. As the score is inherently a discrete variable, we are not gauranteed to find an x_q such that $P(S \geq x_q) = q$ instead we resort to using $x_{q_{is}}$ which is the smallest x such that $P(S \geq x) \geq q$ as determined by importance sampling. We set $q_{is} = P(S \geq x_{q_{is}})$. We then evaluate the saddlepoint approximation in $x_{q_{is}}$, we let q_{saddle} be the saddlepoint approximation to $P(S \geq x_{q_{is}})$. The relative error is then:

$$E = \frac{q_{saddle} - q_{is}}{q_{is}} \tag{S3}$$

We take the average of the absolute value of the relative errors for each quantile in a geometric progression from 0.1 to 0.001, i.e. (0.1, 0.07502, 0.05624, 0.04218, 0.03163, 0.02371, 0.01778, 0.01334, 0.01, 0.007502, 0.005624, 0.004218, 0.003163, 0.002371, 0.001778, 0.001334, 0.001). Generally, the SA is known to have a small relative error for very rary events, however SA is developed for continuous variables, our score is inherently discrete. The SA can be corrected in the case that the variable takes values on a lattice. The correction factor is

$$K(\theta, \alpha) = \frac{\alpha\,|\theta|}{1 - \exp\left(-\alpha\,|\theta|\right)}, \tag{S4}$$

where α is distance between two consecutive points in the lattice. Our scores will typically not take values on a lattice, still the form of the correction factor suggests that we will typically underestimate the tail probability and furthermore this will be more pronounced for extreme scores. For the cases we investigated this effect was relatively modest(see fig. S3).

We measure the degree of independency between the contributions from each factor by the ratio of the variance of the score and the sum of variances from each factor.

$$VR = \frac{\mathbb{V}\left[\sum_{a \in \mathcal{A}} S_a(X_a)\right]}{\sum_{a \in \mathcal{A}} \mathbb{V}\left[S_a(X_a)\right]}$$

By varying p we obtain a range of variance ratios.

We investigate how different parameters affect the error: The size of the graph, N, the complexity of the graph, Δ and the degree of independence between the nodes, VR. As expected the quality of the approximation improves with the size of the graph N under all scenarios (Fig. 6A). We observe that the relative error is lowest at variance ratios around 1 (Fig. S4). Making the situation resemble the IID situation. We did speculate that small amounts of negative correlation could make convergence faster, but that did not appear to be the case. Finally we see that the relative error is lower for graphs with low complexity (Fig. S5).

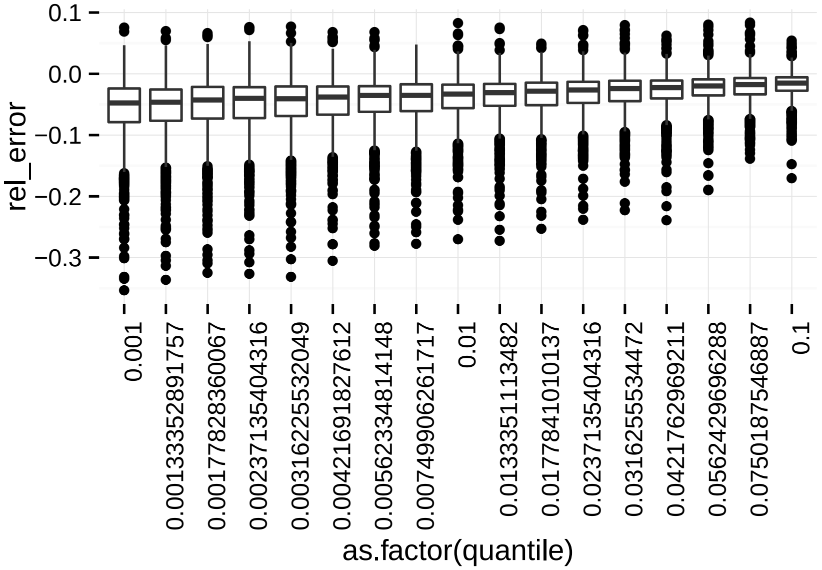

Figure S3: A very slight increase in the relative error of the saddlepoint approximation can be observed as we consider increasingly extreme scores.

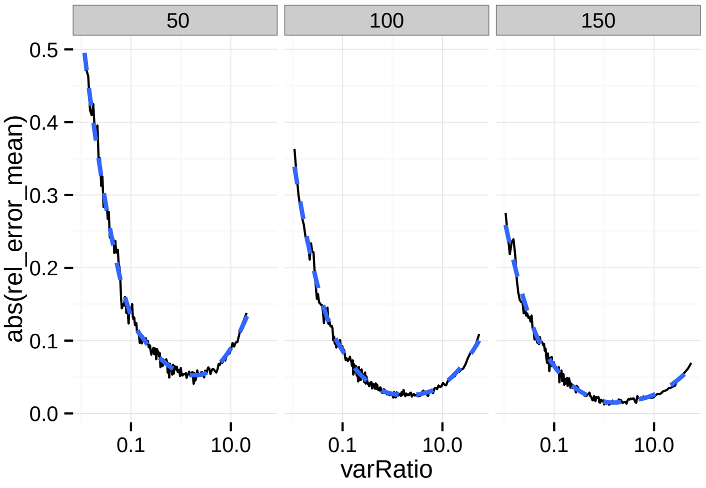

Figure S4: The average absolute relative error in three different scenarios all having degree 1 (i.e. Markov chain) but with 50,100 and 150 variables respectively. We see that the error is lowest for a variance ratio around 1.

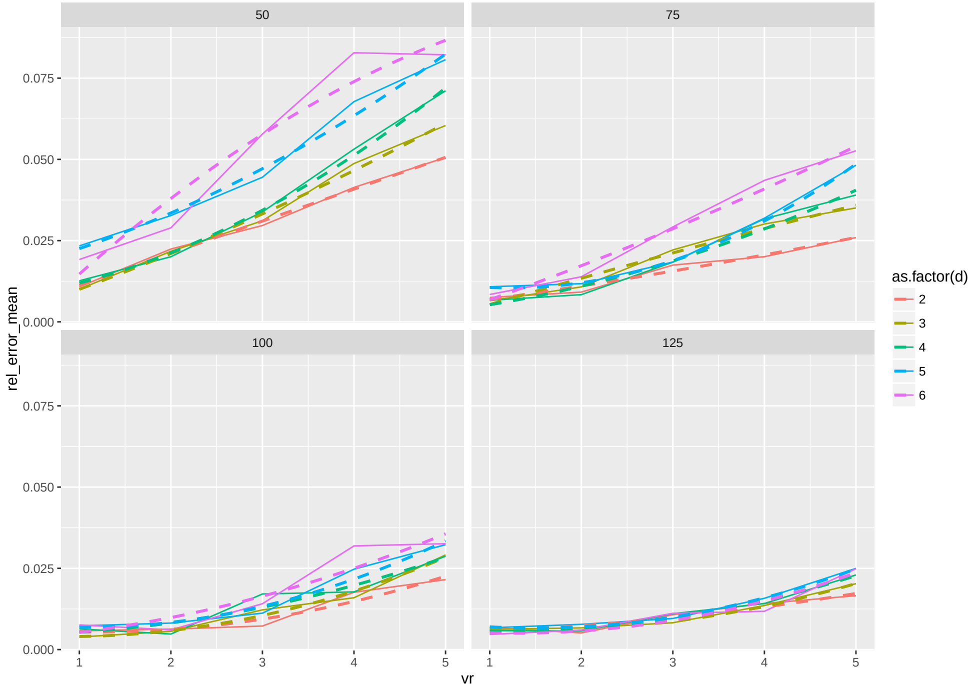

Figure S5: The average absolute relative error as a function of variance ratio (vr) for four different N's stratified into different graph complexities. It is seen the more complex graphs (with high degree) has larger errors.

iii Computational speed

All three methods scale linearly with the number of nodes in the graph. The normal approximation requires us to the run the extended sum-product algorithm once, this is linear in the number of nodes. Similarly the sampling procedure used for importance sampling is basically a depth-first tree-traversal and is also linear in the number of nodes. Finally for the saddlepoint approximation we need to do the extended sum-product algorithm for each iteration in the Newton-Raphson algorithm, it appears that the number of iterations needed is roughly constant. We confirm these theoretical considerations by a benchmark, see Fig. S6.

Another hindrance for a comparison is how to evaluate the significance over a range of values. Normal approximation is near constant in the number of evaluation we want to perform. In importance sampling it is reasonable to use the same sample to evaluate not only in a single point but for multiple points in a neighborhood, making it near constant for multiple evaluations in a neighborhood. For saddlepoint approximation the cost of evaluating consecutive points is linear in the number of points, we can speed up the computation of the saddlepoint (14) by exploiting that κ' is continuous and monotone, making the first evaluation slightly more time consuming (Fig. S7).

Unsurprisingly the computation time for importance sampling is linear in the number of samples we generate (Fig. S8).

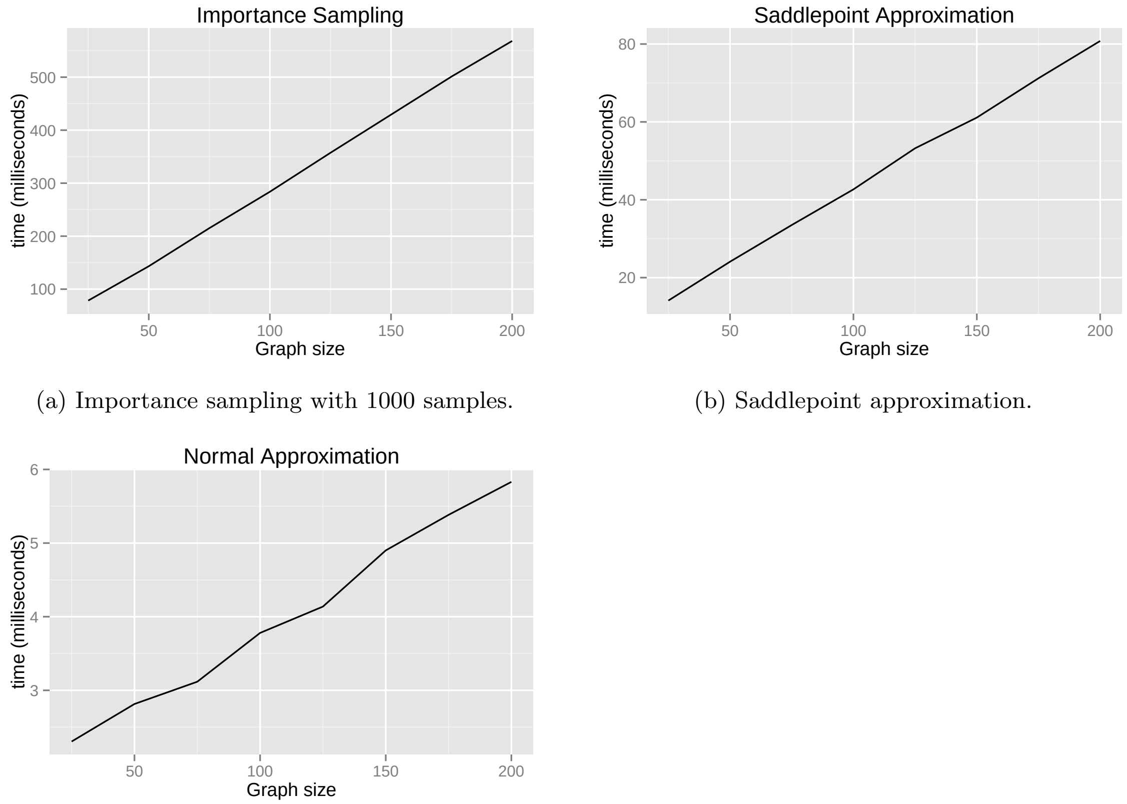

(a) Importance sampling with 1000 samples.

(b) Saddlepoint approximation.

(c) Normal approximation.

Figure S6: The time to evaluate significance for the three different methods as a function of graph size, n. We evaluate only a single point.

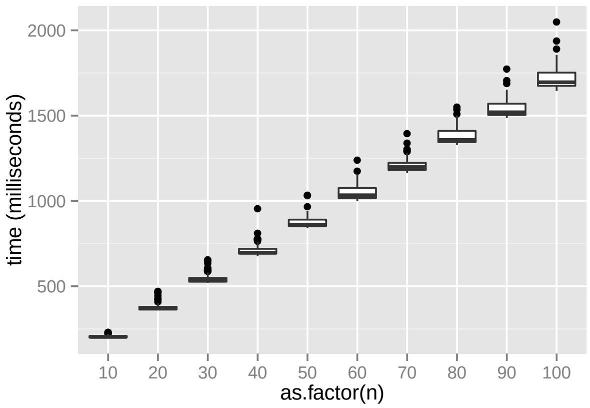

(a) The first evaluation is more expensive as the newton raphson has to home in on the right value, the following points requires 16 ms each, this number will to some extend depend on the spacing between the points we evaluate.

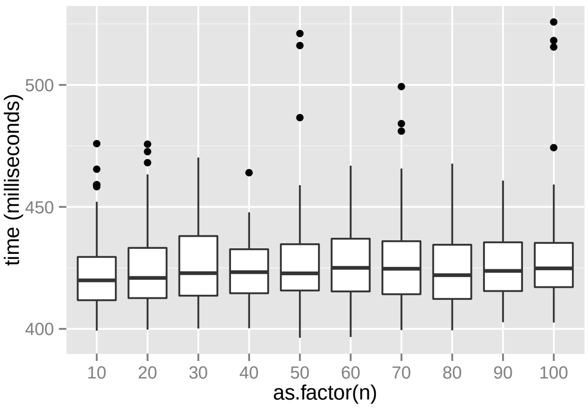

(b) As we have seen the cost is linear in the number of samples we take: 1000 samples take around 420 ms. There is no noticeable increase in time with more consecutive evaluations, if however the points ranges alot we might need to make additional batches of samples with different values of α

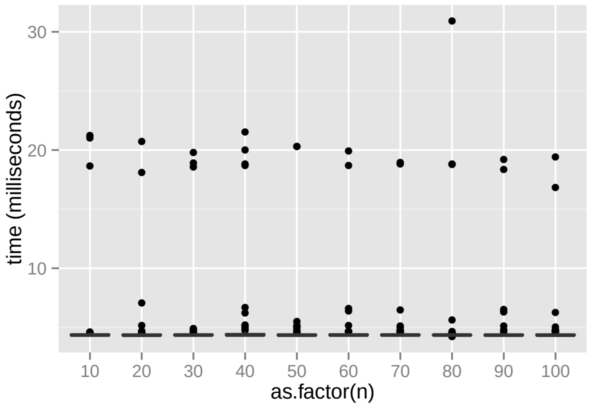

(c) Normal approximation takes around 5 ms.

Figure S7: We compare the computational speed of the tree methods on a graph with 150 variables as the number of points we evaluate, n, increases.

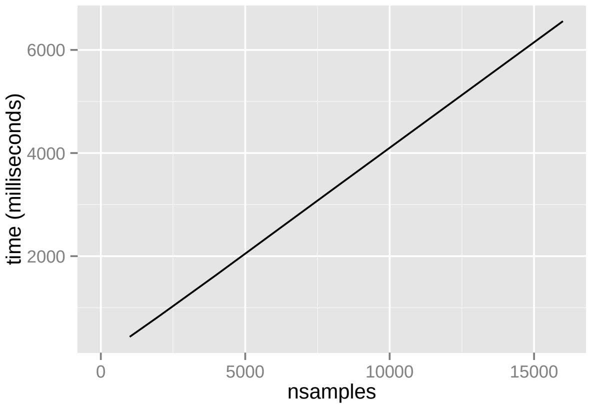

Figure S8: The cost of importance sampling in the same graph with 150 variables as the number of samples increases. The cost is linear in the number of samples we take. 1000 samples take 420 ms.

iv Algorithm for moment calculation in factor graphs

In this section we give the gist of the algorithm involved in computing the first and second moment of the score defined over a factor graph. These moments are neccesary to evaluate eq. (10) and thereby compute the saddlepoint approximation. The algorithm is a generalization of the sum-product algorithm which is based on message passing. The messages have an interpretation as conditional expectations when we consider Bayesian networks. First we explain the main idea with a Markov chain. Second we show how additional terms enter the message computation when a variable node has more than one descendant.

iv.i Markov chain

Consider an inhomogenous Markov chain $X_1, \ldots, X_L$ on a finite state space. The transition probalities are denoted $p^l_{ij} = P(X_{l+1} = j \mid X_l = i)$. We associate a score, $g_l(X_{l+1}, X_l)$, to each transition. The score of a sequence is the sum of these scores

$$S(X_1, \ldots, X_L) = \sum_{l=1}^{L-1} g_l(X_{l+1}, X_l).$$

The calculation of the first and second moment of the score proceeds iteratively by computing

$$\lambda_l(i) = \mathbb{E}\left[\sum_{k=l+1}^{L} g_l(X_k, X_{k-1}) \mid X_l = i\right] \tag{S5}$$

and

$$\gamma_l(i) = \mathbb{E}\left[\left(\sum_{k=l+1}^{L} g_l(X_k, X_{k-1})\right)^2 \mid X_l = i\right]. \tag{S6}$$

Conditioning on X_{l+1}, using the law of total expectation and the Markov property we arrive at a recursion for computing $\lambda_l(i)$

$$\begin{aligned}
\lambda_l(i) &= \sum_j P(X_{l+1} = j \mid X_l = i)\, \mathbb{E}\left[\sum_{k=l+1}^{L} g_l(X_k, X_{k-1}) \mid X_{l+1} = j, X_l = i\right] \\
&= \sum_j p_{ij} \left(g_{l+1}(j,i) + \mathbb{E}\left[\sum_{k=l+2}^{L} g_l(X_k, X_{k-1}) \mid X_{l+1} = j\right]\right) \\
&= \sum_j p_{ij} \left(g_{l+1}(j,i) + \lambda_{l+1}(j)\right).
\end{aligned} \tag{S7}$$

Similarly for the second moment, $\gamma_l(i)$

$$\begin{aligned}
\gamma_l(i) &= \sum_j P(X_{l+1} = j \mid X_l = i)\, \mathbb{E}\,[(\sum_{k=l+1}^{L} g_l(X_k, X_{k-1}))^2 \mid X_{l+1} = j, X_l = i] \\
&= \sum_j p_{ij} \left(g_{l+1}(j,i)^2 + 2g_{l+1}(i,j)\mathbb{E}\left[\sum_{k=l+2}^{L} g_l(X_k, X_{k-1}) \mid X_{l+1} = j\right] + \mathbb{E}\,[(\sum_{k=l+1}^{L} g_l(X_k, X_{k-1}))^2 \mid X_{l+1} = j]\right) \\
&= \sum_j p_{ij} \left(g_{l+1}(j,i)^2 + 2g_{l+1}(j,i)\lambda_{l+1}(j) + \gamma_{l+1}(j)\right).
\end{aligned} \tag{S8}$$

The recursions are initalized with $\gamma_L(i) = 0$ and $\lambda_L(i) = 0$.

Knowing the initial distribution, $\pi_i = P(X_1 = i)$, we can calculate the two moments

$$\begin{aligned}\mathbb{E}\left[S(X_1,\dots,X_L)\right] =& \mathbb{E}\left[\sum_{l=1}^{L-1} g_l(X_{l+1},X_l)\right]\\ =& \sum_i P(X_1=i)\,\mathbb{E}\left[\sum_{l=1}^{L-1} g_l(X_{l+1},X_l)\mid X_1=i\right]\\ =& \sum_i \pi_i\lambda_1(i)\end{aligned}$$

and

$$\begin{aligned}\mathbb{E}\left[S(X_1,\dots,X_L)^2\right] =& \mathbb{E}\left[\left(\sum_{l=1}^{L-1} g_l(X_{l+1},X_l)\right)^2\right]\\ =& \sum_i P(X_1=i)\,\mathbb{E}\left[\left(\sum_{l=1}^{L-1} g_l(X_{l+1},X_l)\right)^2\mid X_1=i\right]\\ =& \sum_i \pi_i\gamma_1(i).\end{aligned}$$

iv.ii Bayesian Network

For the Markov chain we can think of λ_l and γ_l as a pair of messages being passed from X_l to X_{l-1} containing the conditional first and second order moment of the score contributions for descendants of X_l in a Bayesian network. We now consider the case where a variable node X has two descendants Y and Z, suppose that Y and Z are conditionally independent given X (see Fig. S9). Furthermore Y and Z both has descendants H and I that are conditionally independent of X given Y and Z respectively, i.e.

$$\begin{aligned}Y &\perp\!\!\!\perp Z\mid X\\ X &\perp\!\!\!\perp H\mid Y\\ X &\perp\!\!\!\perp I\mid Z.\end{aligned}$$

Again introduce a score, that is a sum over variables neighbouring the same edge in the Bayesian network

$$S(X,Y,Z,H,I) = g_{XY}(X,Y) + g_{XZ}(X,Z) + g_{YH}(Y,H) + g_{ZI}(Z,I)$$

Suppose that we have already calculated

$$\lambda_{Y\to X}(y) = \mathbb{E}\left[g_{YH}(Y,H)\mid Y=y\right]$$

and

$$\gamma_{Y\to X}(y) = \mathbb{E}\left[g_{YH}(Y,H)^2\mid Y=y\right].$$

and the corresponding terms for $Z\to X$.

We can then calculate the messages from variable node X, i.e. the first and second moment of the score conditioned on X. Again we obtain recursions using the incoming messages from Y and Z.

$$\begin{aligned}\lambda_{X\to\cdot}(x) =& \sum_y\sum_z P(Y=y|X=x)P(Z=z|X=x)\mathbb{E}\left[g_{XY}(X,Y)+g_{XZ}(X,Z)+g_{YH}(Y,H)+g_{ZI}(Z,I)|X=x,Y=y,Z=z\right]\\ =& \sum_y\sum_z P(Y=y|X=x)P(Z=z|X=x)\Big(g_{XY}(x,y)+g_{XZ}(x,z)+\lambda_{Y\to X}(y)+\lambda_{Z\to X}(z)\Big)\end{aligned}$$

$$\gamma_{X\to\cdot}(x) = \sum_y \sum_z P(Y=y|X=x)P(Z=z|X=x)\mathbb{E}\left[(g_{XY}(X,Y)+g_{XZ}(X,Z)+g_{YH}(Y,H)+g_{ZI}(Z,I))^2\,|X=x,Y=y,Z=z\right]$$
$$= \sum_y \sum_z P(Y=y|X=x)P(Z=z|X=x)\Big(g_{XY}(x,y)^2 + g_{XZ}(x,z)^2 + \gamma_{Y\to X}(y) + \gamma_{Z\to X}(z) +$$
$$2g_{XY}(x,y)g_{XZ}(x,z) + 2\lambda_{Y\to X}(y)\lambda_{Z\to X}(z) +$$
$$2g_{XY}(x,y)\lambda_{Y\to X}(y) + 2g_{XY}(x,y)\lambda_{Z\to X}(z) + 2g_{XZ}(x,z)\lambda_{Z\to X}(z) + 2g_{XZ}(x,z)\lambda_{Y\to X}(y)\Big).$$

Notice that additional quadratic terms enter when a variable has more than one descendant. We encourage the reader to think of I and H and $g_{YH}(Y,H)$ and $g_{ZI}(Z,I)$ as contributions from a larger subtree.

We have here sketched the algorithm in the case where messages are passed backwards in a Bayesian network. The general algorithm operates on factor graphs and the messages can not be interpreted as conditional expectations. Still this example contains the main ideas used in the general case. We are preparing a manuscript on the general algorithm, the manuscript has been made available to the reviewers and is available upon request.

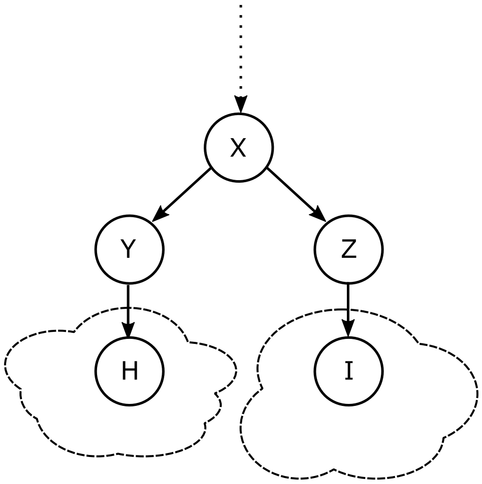

Figure S9: A section of a Bayesian network. We want to compute the first and second moment of the sum of score contributions in the subtree at X conditional on the value of X.

v dgRaph

Analysis were carried out using an R-package called dgRaph[1], that we have developed. Here follows a tutorial-style vignette, this vignette is also included with the package, where potential changes to the interface will be reflected. For each of the examples in the main text we have included vignettes describing the analysis.

Let us start with an example of how to specify a factor graph:

```
library(dgRaph)
varDim <- rep(4,5)
facPot <- list(multinomialPotential(c(1,4)),
               multinomialPotential(c(4,4)))
facNbs <- list(c(1L),
               c(1L,2L),
               c(1L,3L),
               c(3L,4L),
               c(3L,5L))
potMap <- c(1,2,2,2,2)
facNames <- c("P",rep("I",4))
varNames <- c("Do","Re","Mi","Fa","Sol")
mydfg <- dfg(varDim, facPot, facNbs, potMap, varNames, facNames)
```

We will give a description of each argument below. The factor graph object can be plotted with the `plot` function:

```
plot(mydfg, layout = layout.reingold.tilford)
```

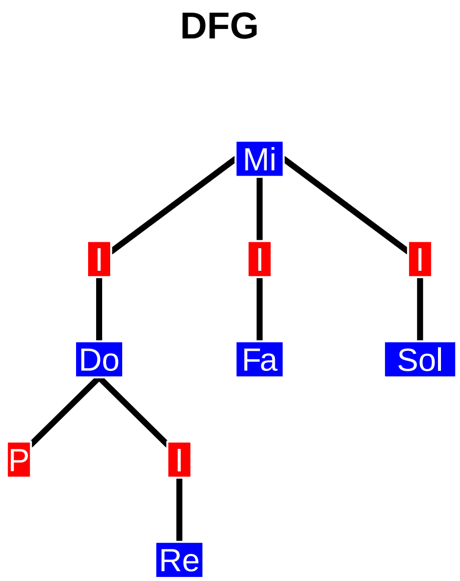

`varDim` holds the number of states for each variable. The support for continuous variables is mediated through discretisation, the number of states for a continuous variable is the number of bins it is discretised into

```
varDim
```

```
## [1] 4 4 4 4 4
```

`facPot` is a list of matrices holding factor potentials. The list does not have to have the same number of elements as there are factor nodes; using the `potMap` argument the same potential can be used for multiple factors.

[1]See `https://github.com/TobiasMadsen/dgRaph`

```r
facPot
```

```
## [[1]]
## $mat
##           [,1]      [,2]      [,3]      [,4]
## [1,] 0.2871007 0.3075834 0.1511901 0.2541258
##
## $param
## $param$independent
## [1] FALSE
##
## $param$pc
## [1] 0
##
## $param$noopt
## [1] FALSE
##
##
## attr(,"class")
## [1] "multinomialPotential" "potential"
##
## [[2]]
## $mat
##           [,1]       [,2]      [,3]      [,4]
## [1,] 0.3061806 0.21942087 0.2512377 0.2231608
## [2,] 0.5112009 0.22540541 0.1254869 0.1379069
## [3,] 0.3094722 0.09863342 0.3447191 0.2471753
## [4,] 0.1217527 0.04599736 0.4306187 0.4016312
##
## $param
## $param$independent
## [1] FALSE
##
## $param$pc
## [1] 0
##
## $param$noopt
## [1] FALSE
##
##
## attr(,"class")
## [1] "multinomialPotential" "potential"
```

`dgRaph` supplies a number of functions that generates potentials for common distri- butions. By default they are initialized to reasonable random values which is useful for EM-training, it is of course also possible to provide parameter values.

```r
multinomialPotential(dim = c(1, 5))
normalPotential(dim = c(1, 100))
betaPotential(dim = c(1, 100), range = c(0,1))
linregPotential(dim = c(100, 100))
```

`facNbs` is a list of neighbours for each factor. The corresponding factor potential should have as many rows as the first neighbour and as many columns as the second neighbour.

```
facNbs
```

```
## [[1]]
## [1] 1
##
## [[2]]
## [1] 1 2
##
## [[3]]
## [1] 1 3
##
## [[4]]
## [1] 3 4
##
## [[5]]
## [1] 3 5
```

Finally `potMap` is a vector providing the mapping between factors and potentials. `potMap[i]` is the number of the potential corresponding to the i'th factor.

```
potMap
```

```
## [1] 1 2 2 2 2
```

v.i Functionality

A number of methods takes data as input. The accepted data format is a matrix or data frame with one column for each variable. Each row corresponds to a single observation. An observation is a vector of integers indicating the states/bins of the variables, missing data or latent variables are indicated by `NA`. We have considered matching variables by column name, but this has not yet been implemented.

```
data <- matrix(c(1,2,3,4,1,
                 NA,NA,2,1,NA,
                 2,1,NA,NA,3), 3, 5, byrow = T)
```

`likelihood` takes data and a dgRaph object and outputs a vector of likelihoods, one for each observation.

```
likelihood(data, mydfg)
```

```
## [1] 0.001210664 0.081244312 0.040781171
```

`mps` calculates the most probable states, that is an assignment of the unobserved variables that maximises the complete likelihood function.

```
data <- matrix(c(1,2,3,4,1,
                 NA,NA,2,1,NA,
                 2,1,NA,NA,3), 3, 5, byrow = T)
mps(data, mydfg)
```

```
##   Do Re Mi Fa Sol
## 1  1  2  3  4   1
## 2  2  1  2  1   1
## 3  2  1  1  1   3
```

facExpectations is the work horse of the EM-algorithm, providing the posterior distributions for the variables neighbouring a factor. These posterior distributions are then summed up over all observations. If we have observations $X_1, \ldots, X_N$, the expectation counts for factor a is the following table

$$t(x_a) = \sum_{i=1}^{N} \mathbb{E}\left[\mathbb{I}(X_a = x_a) \mid X_i\right]$$

Obtaining these counts constitutes the E-step of the EM-algorithm and are used for finding new factor potentials in the M-step. If the same potential is shared by multiple factors, the expectation counts is also summed over these factors.

```
data <- matrix(c(1,2,3,4,1,
                 NA,NA,2,1,NA,
                 2,1,NA,NA,3), 3, 5, byrow = T)
facExpectations(data, mydfg)
```

```
## [[1]]
##          [,1]    [,2]       [,3]      [,4]
## [1,] 1.396379 1.43624 0.09383092 0.0735496
##
## [[2]]
##            [,1]       [,2]      [,3]      [,4]
## [1,] 0.27298094 1.59200784 1.7191847 0.1989631
## [2,] 3.28514697 0.89361690 0.4697582 0.4420746
## [3,] 1.08065354 0.11953641 0.2566250 1.0644180
## [4,] 0.03683227 0.08746458 0.3592368 0.1215002
```

We can simulate observations from a factor graph using the `simulate` function

```
simulate(mydfg, 6)
```

```
##   Do Re Mi Fa Sol
## 1  4  4  3  1   3
## 2  1  4  3  4   1
## 3  2  1  1  4   1
## 4  3  3  1  1   4
## 5  4  4  4  3   3
## 6  2  1  2  3   2
```

The output is a data frame in the same format that is used for data-input. We can also calculate expectations of the form $\mathbb{E}\left[\sum_{a \in \mathcal{A}} g_a(x_a)\right]$. The functions $\{g_a\}_{a \in \mathcal{A}}$ should be given as a list of matrices, `facScore`, with same length and dimensions as the potentials.

```
facScore <- list(matrix(0,1,4), diag(4))
expect(mydfg, facScore)
```

```
## Likelihood     Expect
##   1.000000   1.277457
```

We can calculate the Kullback-Leibler divergence between two factor graphs with the same structure, by which we mean that they share the same set of variables and if there is a factor a with neighbour variables $Ne(a)$ in the first graph then there exist a factor b with the same set of neighbours in the second graph. The factors do not have to appear in the same order and factors that are shared in the first graph do not have to be shared in the second and vice versa.

```r
# Make a second factor graph with different potentials
mydfg2 <- mydfg
potentials(mydfg2) <- list(multinomialPotential(c(1,4)),
                           multinomialPotential(c(4,4)))

# Calculate kl
kl(dfg1 = mydfg, dfg2 = mydfg2)
```

```
##       kl
## 2.466517
```

v.ii Tail approximations

Consider two factor graph models P_{bg} and P_{fg}, where we think of P_{bg} as a background model and P_{fg} as a foreground model. We can define the score for an observation as

$$S(X) = \log \frac{P_{fg}(X)}{P_{bg}(X)}. \tag{S9}$$

We are interested in evaluating, $P_{bg}(S(X) > s)$, i.e. the tail of the score-distribution under the background model. We have implemented two approximation methods for this problem, `tailIS` uses importance sampling, whereas `tailSaddle` uses a saddle point approximation.

```r
dfis <- tailIS(x = seq(0,3,0.001), n = 10000, alpha = 0.5, dfg1 = mydfg, dfg2 = mydfg2)
dfsaddle <- tailSaddle(x = seq(0,3,0.001), dfg1 = mydfg, dfg2 = mydfg2)
```

We can plot the result of the two methods together. The sampling based approach is plotted with confidence bands around it and the saddle point approximation is the dashed green line.

```r
library(ggplot2)
ggplot(dfis, aes(x=x,y=p)) + geom_line() + theme_bw() +
  theme(axis.line = element_line(colour = "black"),
        #panel.grid.major = element_blank(),
        panel.grid.minor = element_blank(),
        panel.border = element_blank(),
        panel.background = element_blank()) +
  geom_ribbon(aes(ymin=pmax(low,0.0001),ymax=high),alpha=0.3,fill="blue") +
  annotation_logticks(sides="l") +
  geom_line(data=dfsaddle, aes(y=p), colour = "darkgreen", size = 0.8, linetype = "dashed") +
  ggtitle("Tail approximations")
```

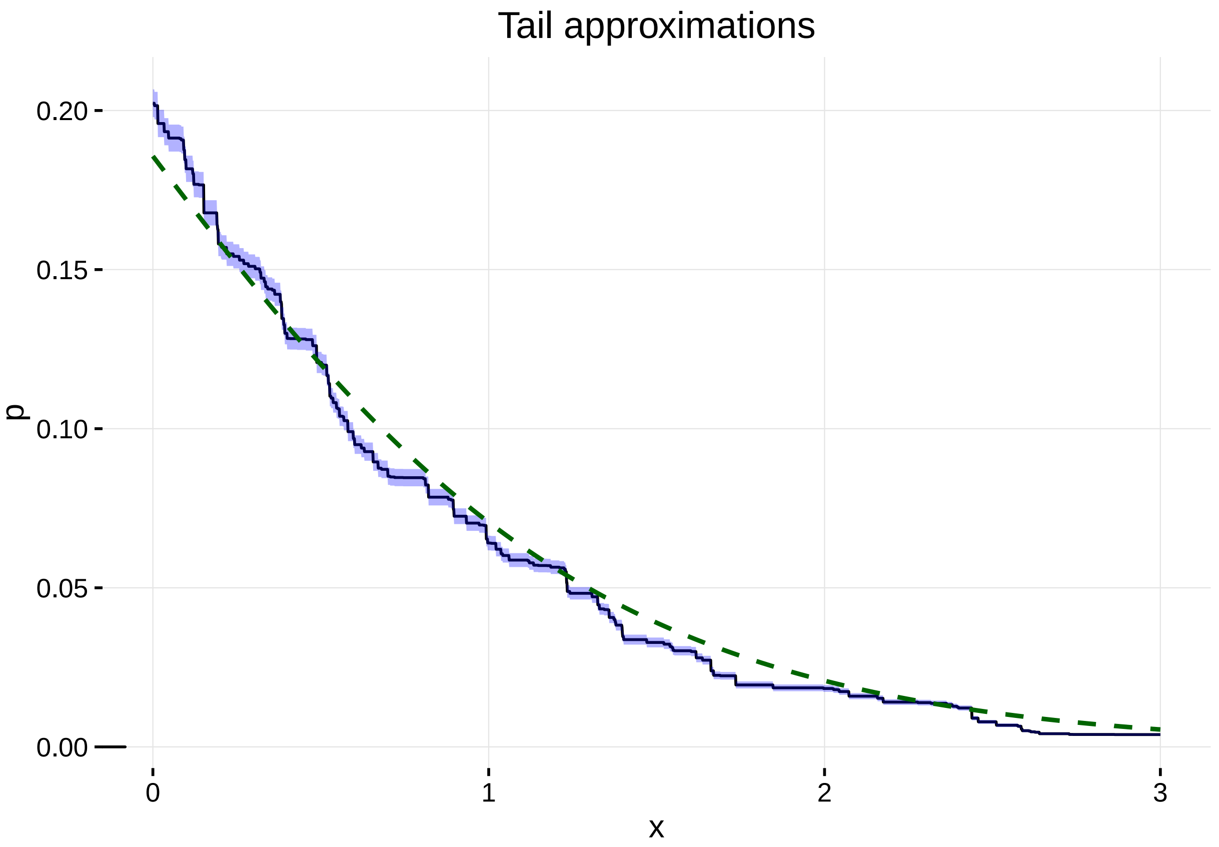

v.iii EM-algorithm

If our factor graph is parameterised by $\theta = (\theta_a)_{a \in \mathcal{A}}$, we can write

$$l_0(\theta; X) = \log L_0(\theta; X) = \log \prod_{a \in \mathcal{A}} f_a(X_a, \theta) = \sum_{a \in \mathcal{A}} \log f_a(X_a, \theta),$$

assuming(!) that the graph is normalized for all θ. The M-step of the EM-algorithm(see p. 276 of *The Elements of Statistical Learning*) consist of maximizing

$$\begin{aligned} \mathbb{E}\left[l_0(\theta'; X^{full}) \mid X^{obs}, \theta\right] =& \mathbb{E}\left[\sum_{a \in \mathcal{A}} \log f_a(X_a, \theta'_a) \mid X^{obs}, \theta\right] \\ =& \sum_{a \in \mathcal{A}} \mathbb{E}\left[\log f_a(X_a, \theta'_a) \mid X^{obs}, \theta\right], \end{aligned}$$

with respect to θ', where the expectation is taken over the distribution of the full data induced by θ and conditioned on the observed data. We see that we can optimise each potential individually, and the expectation counts are the statistics we need.

To each factor we associate an optimisation function, which update the potential in the M-step of the EM-algorithm. We provide predefined functions for performing this update, corresponding to the distribution the potential encodes, e.g. normal distribution or multinomial distribution. At the same time we allow for implementing custom optimisation functions, thus having a high degree of flexibility.

The EM-algorithm is implemented in the `train` function. The function takes a factor graph object and data to learn the parameters from:

```r
head(dftrain)
```

```
##   Do Re Mi Fa Sol
## 1 NA  1  2  2   2
## 2 NA  3  3  3   2
## 3 NA  4  1  1   1
## 4 NA  3  1  4   3
## 5 NA  4  4  4   4
## 6 NA  1  2  2   3
```

The `train` function outputs a new factor graph object with optimised potentials.

```r
mydfgtrained <- train(data = dftrain,
                      dfg = mydfg,
                      verbose = T,
                      iter.max = 1000,
                      threshold = 1e-5)
```

```
## Training...
## Iterations:...........................
## EM-algorithm converged after 28 iterations
## Likelihood: -4401.177
```

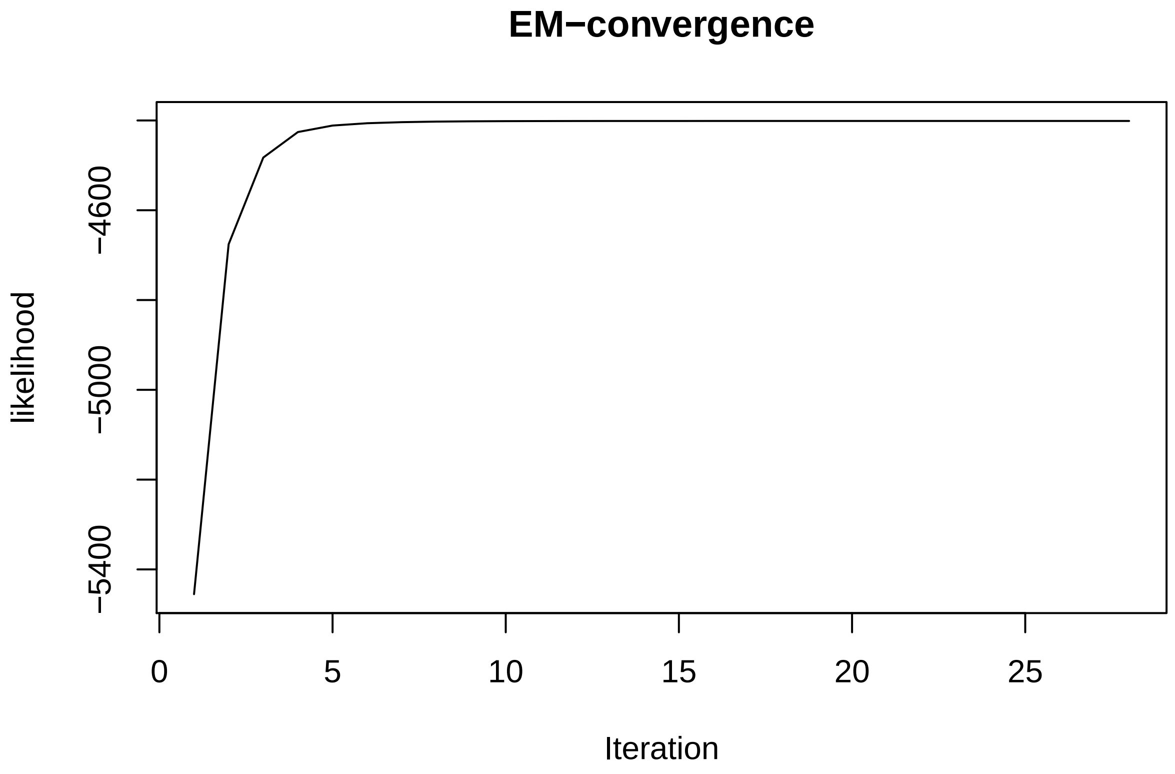

vi Example 1: The Poisson-Binomial distribution

The Poisson-binomial distribution is defined as a sum of indepedent bernouilli variables. Let $p_1, \ldots, p_N$ be a set of probabilities, $\{Y_n\}_{n=1}^N$ be independent and $Y_n \sim \text{Binom}(p_n, 1)$. Then $X = \sum_{n=1}^N Y_n$ has a Poisson-Binomial distribution.

The R-package `poibin` [3] computes the density and cumulative distribution function for the Poisson-Binomial distribution using Fast Fourier Transform (FFT). [3] does not provide an analysis of the complexity of their algorithm, even if FFT has complexity $\mathcal{O}(N \log N)$, there is a preprocessing step in the algorithm taking $\mathcal{O}(N^2)$. Constrastingly, the saddlepoint approximation scales linearly with N, having complexity $\mathcal{O}$(N).

The binomial distribution arises as the special case where $p_n = p \ \forall n \in \{1, \ldots N\}$. We benchmarked (table 1) the saddlepoint approximation and the `poibin` package using different N's keeping the mean at 10 and evaluating the tail probability, $P(X > x)$ for $x \in \{1, \ldots, 50\}$.

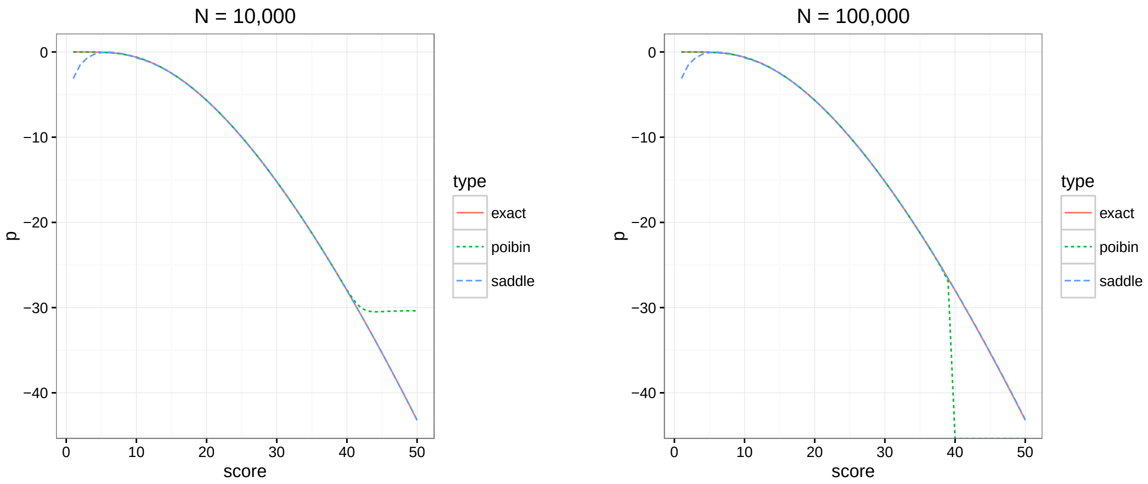

Figure S10: We compute the tail of the Poisson-binomial distribution where $p_i = p$, and thus reduces to a binomial distribution. p is set to $\frac{10}{N}$ such that the mean is 10. We compare the saddlepoint method, the DFT-CF method and R's built-in cummulative distribution function. This confirms that the DFT-CF method experiences underflow for small p-values.

N	Type	min	lq	mean	median	uq	max	neval
1000	poibin	13.49	13.54	14.42	14.06	15.32	16.50	20
1000	saddle	25.43	25.75	27.53	27.27	28.96	31.72	20
2000	poibin	53.55	53.83	55.42	55.68	56.61	57.84	20
2000	saddle	47.23	49.02	51.08	50.74	53.15	56.62	20
3000	poibin	124.98	126.99	130.34	128.60	132.94	139.98	20
3000	saddle	68.96	69.73	72.92	72.23	74.35	81.15	20
4000	poibin	224.27	227.51	234.08	230.91	238.48	259.01	20
4000	saddle	89.38	91.97	94.73	93.38	96.31	107.87	20
5000	poibin	341.04	348.13	356.64	351.95	368.85	377.34	20
5000	saddle	110.94	114.12	119.71	117.16	124.46	143.02	20
6000	poibin	489.96	494.74	508.27	500.13	523.92	544.88	20
6000	saddle	132.52	138.25	143.56	140.39	149.86	159.23	20
7000	poibin	686.60	694.14	706.14	703.90	708.70	762.33	20
7000	saddle	154.68	159.19	164.25	161.64	167.74	177.80	20
8000	poibin	864.03	876.36	895.28	884.60	910.79	956.05	20
8000	saddle	176.57	181.70	194.17	191.93	203.95	221.21	20
9000	poibin	1140.25	1153.76	1180.50	1169.91	1215.55	1240.78	20
9000	saddle	199.20	201.04	208.75	207.81	212.85	226.56	20
10000	poibin	1360.09	1377.10	1401.26	1391.02	1411.91	1486.86	20
10000	saddle	219.48	228.65	236.66	236.61	246.24	255.91	20

Table 1: The computation time for each of the two methods for different lengths N. We replicate each computation 20 times in random order using the `microbenchmark` R-package and report minimum, maximum, lower quantile, upper quantile, mean and median running time. A plot is shown in Fig. 2 in the main text.

vii Poisson-Binomial Vignette

```
library(poibin)
library(dgRaph)
library(ggplot2)
```

The dgRaph package has a specialized function for the Poisson-binomial model and the generalization where a score is assigned to each variable. I.e. assume $X_1, \ldots X_N$ are independent Bernouilli trials where $X_i \sim \text{Bernouilli}(p_i)$. We look at the score

$$S = \sum_{i=1}^{N} s_i X_i$$

and evaluate the probability $P(S \geq t)$. First consider the simple case of a binomial distribution where $s_i = 1$ and $p_i = p$.

```
N <- 10000
p <- 0.005
tail_p_SA <- sapply(51:101, poibinSA, p = rep(p, N), log = T)
```

Evaluate the same probabilities using the built in binomial CDF function.

```
tail_p_binom <- pbinom(50:100, N, p, lower.tail = F, log.p = T)
```

Plot the two against each other

```
df <- data.frame(p = c(tail_p_SA, tail_p_binom), x = 50:100,
                 type = c(rep("SA", 51), rep("Built-in", 51)) )
ggplot(df, aes(y = p, x = x, colour = type)) + geom_line()
```

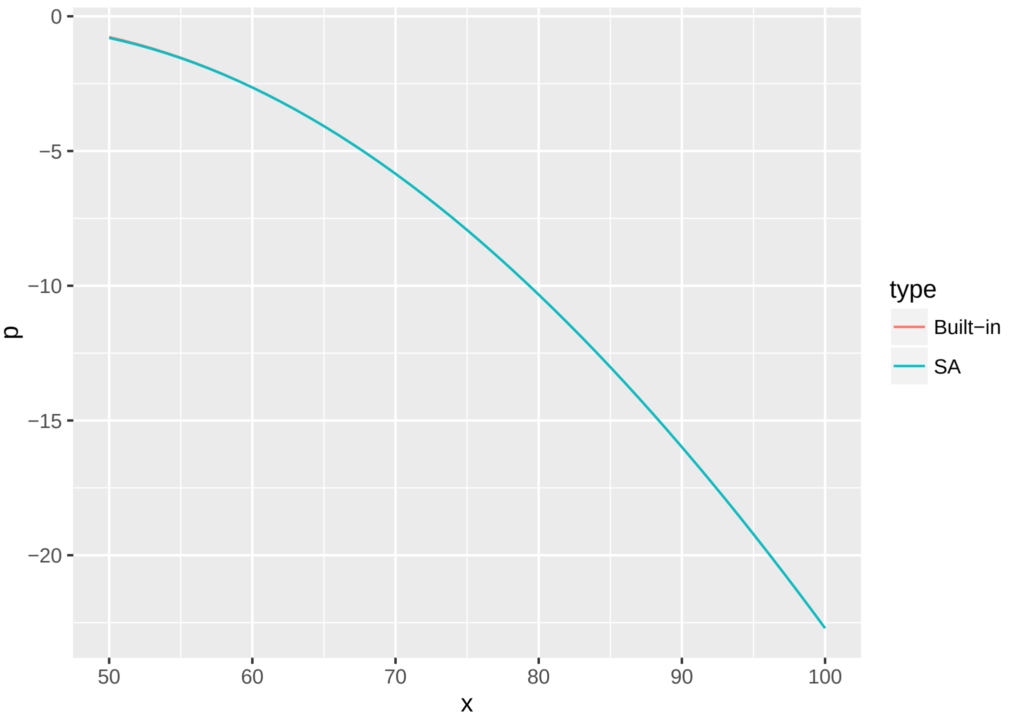

Now we look at the Poisson-Binomial model. Where the p_i's are not identical.

```r
N <- 1000
p <- rbeta(N, 1, 100)
```

Tail distribution

```r
tail_p_SA <- sapply(1:50, poibinSA, p = p)
tail_p_poibin <- log1p(-ppoibin(1:50-1, pp = p))
```

We can again compare the estimates

```r
df <- data.frame(p = c(tail_p_SA, tail_p_poibin), x = 1:50,
                 type = c(rep("SA", 50), rep("DFT-CF", 50)) )
ggplot(df, aes(y = p, x = x, colour = type)) + geom_line()
```

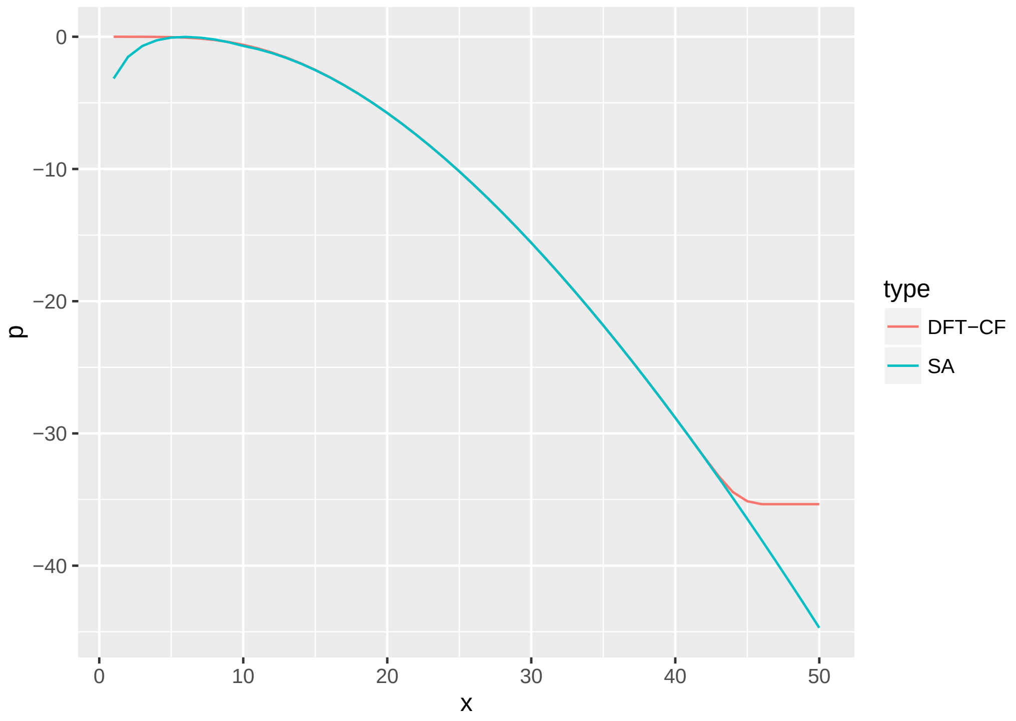

viii Example 2: Position Weight Matrices

We benchmarked the exact p-value computation implemented in the `TFMPvalue` R-package (Fig. S11). The exact calculation has exponential computational complexity, whereas SA has linear running time. Both methods evaluate a single point at a time.

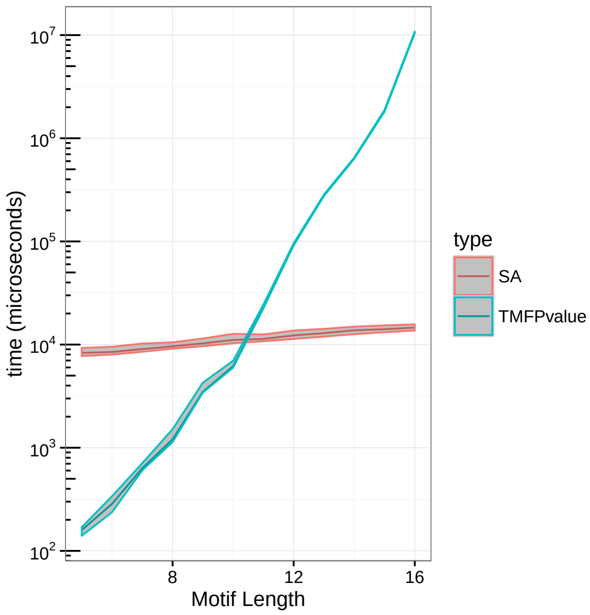

Figure S11

We have included the PWM analysis as a vignette to the ‘dgRaph‘ R-package.

We also benchmarked the non-exact TFMPvalue implementation where the the scores are rounded (Fig. S12). We simulated a motif where the frequencies at each position was drawn from a Dirichlet-distribution, Dirichlet$(0.5, 0.5, 0.5, 0.5)$. The frequencies was transformed into scores using log2-fold enrichment. The maximal score was 1.51.

The running time of the algorithm depends on how coarse the discretization is. Here we discretized to 10^{-3}. As the non-exact TFMP-value algorithm is not implented in the corresponding R-package, we ran the software command line with the following arguments:

```
./TFMpvalue-distrib -a 0.29 -c 0.21 -g 0.21 -t 0.29 -m benchmark/tmp.pwm -w -s -200 -S 100
-G 0.001
```

Two important factors must be mentioned when comparing the running times of TFMP non-exact to TFMP exact and SA. First, the coarseness of the discretization influences the running time. Second, TFMP non-exact computes estimates of the whole distribution of the score.

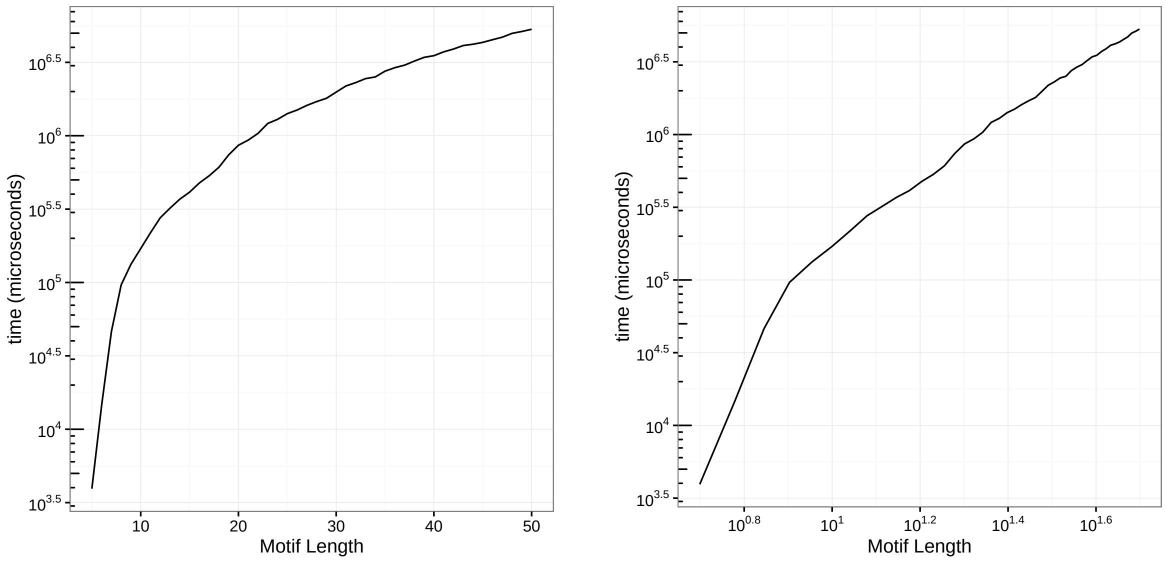

Figure S12: The running time of non-exact TFMP plotted against motif length. The running-time of the non-exact TFMP is clearly sub-exponential and the plot on log-log scale indicates that it has quadratic running time consistent with the theory.

ix PWM Vignette

```
library(dgRaph)
library(ggplot2)
```

The CTCF binding motif is obtained from the Jaspar database (ID: MA0139.1).

```
PFM
```

```
##      [,1] [,2] [,3] [,4] [,5] [,6] [,7] [,8] [,9] [,10] [,11] [,12] [,13]
## [1,]   87  167  281   56    8  744   40  107  851     5   333    54    12
## [2,]  291  145   49  800  903   13  528  433   11     0     3    12     0
## [3,]   76  414  449   21    0   65  334   48   32   903   566   504   890
## [4,]  459  187  134   36    2   91   11  324   18     3     9   341     8
##      [,14] [,15] [,16] [,17] [,18] [,19]
## [1,]    56   104   372    82   117   402
## [2,]     8   733    13   482   322   181
## [3,]   775     5   507   307    73   266
## [4,]    71    67    17    37   396    59
```

After adding a pseudocount we convert the frequencies to relative freqecies (PPM) and position weight matrices (PWM).

```
PPM <- scale(PFM+1, center = FALSE, scale = colSums(PFM+1))
row.names(PPM) <- c('A','C','G','T')

PWM <- log(t(scale(t(PPM), center = FALSE, scale = c(0.29,0.21,0.21,0.29))))
```

We build the PWM model as well as a background model using the `dgRaph` package.

```
varDim <- rep(4, 19)
facPot <- lapply(1:19, function(i){matrix(PPM[,i], 1, 4)})
facPotBg <- list(matrix(c(0.29, 0.21, 0.21, 0.29), 1, 4))
facNbs <- lapply(1:19, function(i){i})
pwm_dfg <- dfg(varDim, facPot, facNbs)
bg_dfg <- dfg(varDim, facPotBg, facNbs, potMap = rep(1, 19))
```

Next evaluate the significance over a range of scores from 0 to 10 using both Saddlepoint Approximation and Importance Sampling.

```
score_dist_saddle <- tailSaddle(seq(0, 10, 0.01), bg_dfg, pwm_dfg)
score_dist_saddle$method <- "SA"
score_dist_is <- tailIS(seq(0, 10, 0.01),
                        n = 1000,
                        alpha = 0.5,
                        dfg1 = bg_dfg, dfg2 = pwm_dfg) %>%
  as.data.frame()
score_dist_is$method <- "IS"
```

Plot the two estimates against each other.

```
plot_df <- rbind(score_dist_saddle[,c("x","p","method")],
                 score_dist_is[,c("x","p","method")])

ggplot(plot_df, aes(x = x, y = p, colour = method)) +
  geom_line() +  scale_y_log10() + theme_bw()
```

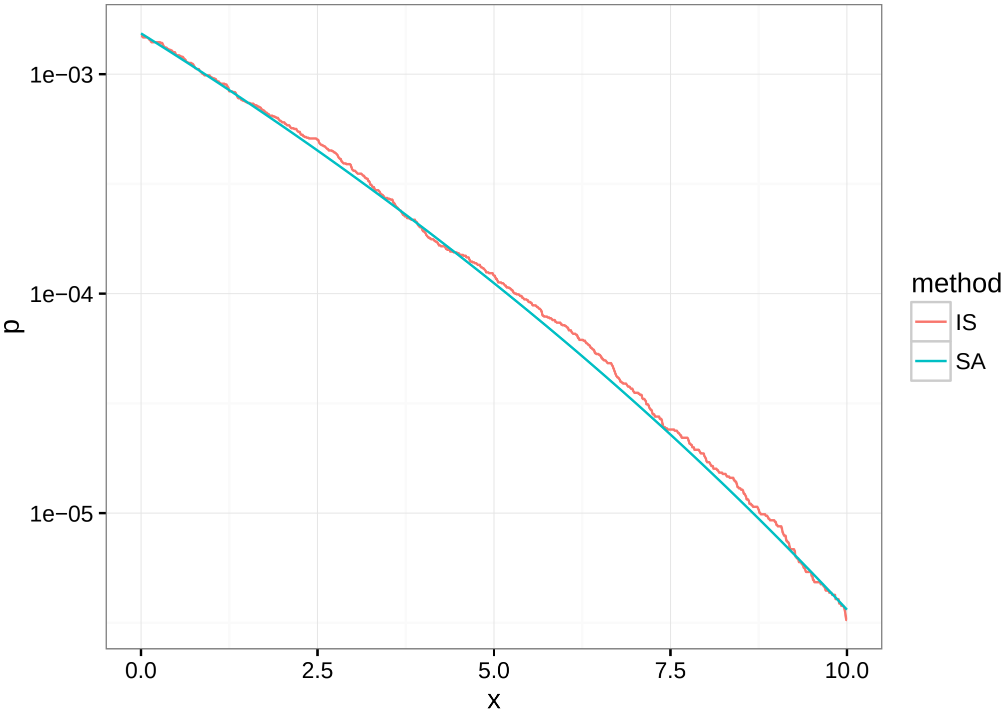
1e−03
1e−04
1e−05
p
0.0
2.5
5.0
7.5
10.0
x
method
IS
SA

x Example 3: BaMM motifs

We obtain a second order Markov motif model using the BaMM software [7] [2]. The software discovers the motifs in sequence proximal to ChIP-seq peaks. We obtain ChIP-seq peaks from the ENCODE 2012 data freeze called using the IDR framework[3] for the CTCF motif in MCF-7 cell lines. The peaks are obtained from the UCSC genome browser [4]. We simply use `awk` to select +/- 100 bp around a ChIP-seq peak site. We extract the corresponding DNA sequences using `twoBitToFa`[5] and the hg19 reference genome sequence. We finally run the BaMM software with the following options:

```
BaMMmotif Bamms/ Sequence/CTCF.fa --saveBaMMs --verbose --reverseComp --XX-localization \
--XX-localizationRanking --XX-K 2 --XX-mergeMotifsThreshold LOW
```

The pipeline is chosen to reproduce the analysis performed by Sierbert and colleagues as close as possible. However for illustrative purposes only one motif is needed, but the analysis extends to the other motifs as well.

To evaluate the significance of the maximum log-odds score obtained by offsetting the motif model along a longer sequence, we employ the Poisson approximation [1]. For a motif of length l and sequence of length k we can offset the motif to $k-l+1$ positions. For each offset we get a log-odds score S_i for $i = 1, \ldots, k-l+1$. The Poisson approximation states that if k is sufficiently large and the motif is not too low-complex then $X = \sum_{i=1}^{k-l+1} \mathbb{I}(S_i > s)$ will be approximately Poisson distributed with mean $(k-l+1) \cdot P(S > s)$. To get the significance of the maximum of the log-scores over all offsets, we note that

$$\begin{aligned} P(\max_i S_i > s) &= 1 - P(\max_i S_i \leq s) \\ &= 1 - P(X = 0) \\ &\approx 1 - \exp(-(k-l+1)P(S > s)) \\ &= 1 - \exp(-P(S > s))^{k-l+1} \\ &\approx 1 - (1 - P(S > s))^{k-l+1}. \end{aligned} \tag{S10}$$

The last approximation uses $\exp(x) \approx 1 + x$ for small x close to 0. Equation (S10) tells us that we can treat the S_i's as independent events under the afforementioned conditions.

[2] Downloaded from https://github.com/soedinglab/BaMMmotif
[3] https://sites.google.com/site/anshulkundaje/projects/idr
[4] http://hgdownload.cse.ucsc.edu/goldenPath/hg19/encodeDCC/wgEncodeAwgTfbsUniform/
[5] The Jim Kent command line bioinformatics utilities https://github.com/ENCODE-DCC/kentUtils

xi BaMM Vignette

```
library(dgRaph)
library(dplyr)
```

Using the BaMM software[6] we obtain the transition probabilities for a second order Markov chain motif model for the CTCF motif. We then encode the second order Markov chain as a first order Markov chain by compounding variables X_1, (X_1, X_2), (X_2, X_3), …, (X_{15}, X_{16}). We provide the transition probabilites for the Markov chain as a list of matrices, `BammTransProb`. The first matrix is a 1x4 matrix giving the distribution of X_1, the second is a 4x16 matrix giving the conditional distribution of (X_1, X_2) given X_1, the following matrices are 16x16 matrices giving the conditional distribution of (X_{i+1}, X_{i+2}) given (X_i, X_{i+1}).

```
BammTransProb[[1]]
```

```
##           [,1]      [,2]      [,3]      [,4]
## [1,] 0.4050646 0.1028535 0.3727162 0.1193658
```

```
BammTransProb[[2]]
```

```
##            [,1]      [,2]      [,3]      [,4]      [,5]      [,6]
## [1,] 0.03303753 0.2646669 0.6369334 0.0653622 0.0000000 0.0000000
## [2,] 0.00000000 0.0000000 0.0000000 0.0000000 0.2065218 0.3895802
## [3,] 0.00000000 0.0000000 0.0000000 0.0000000 0.0000000 0.0000000
## [4,] 0.00000000 0.0000000 0.0000000 0.0000000 0.0000000 0.0000000
##           [,7]     [,8]       [,9]     [,10]     [,11]      [,12]
## [1,] 0.0000000 0.000000 0.00000000 0.0000000 0.0000000 0.00000000
## [2,] 0.2878351 0.116063 0.00000000 0.0000000 0.0000000 0.00000000
## [3,] 0.0000000 0.000000 0.07693451 0.3764474 0.4699017 0.07671638
## [4,] 0.0000000 0.000000 0.00000000 0.0000000 0.0000000 0.00000000
##           [,13]     [,14]     [,15]      [,16]
## [1,] 0.00000000 0.0000000 0.0000000 0.00000000
## [2,] 0.00000000 0.0000000 0.0000000 0.00000000
## [3,] 0.00000000 0.0000000 0.0000000 0.00000000
## [4,] 0.08459699 0.2819641 0.5550184 0.07842054
```

We also provide the background transition probabilities `BammTransProbBg`. We can now build the foreground and background models in the `dgRaph`.

```
varDim <- c(4, rep(16, 16-1))
facNbs <- c(list(1),lapply(1:15, function(i) c(i, i+1) ))
bamm_dfg <- dfg(varDim, BammTransProb, facNbs)

potMap <- c(1,2,rep(3, 16-2))
bamm_dfg_bg <- dfg(varDim, BammTransProbBg, facNbs, potMap = potMap)
```

Next we evaluate the significance of a range of scores using saddlepoint approximation and importance sampling.

```
score_dist_saddle <- tailSaddle(seq(-15, 5, 0.05), bamm_dfg_bg, bamm_dfg)
score_dist_saddle$method <- "SA"
score_dist_is <- tailIS(seq(-15, 5, 0.05),
                        n = 1000,
```

[6] https://github.com/soedinglab/BaMMmotif

```r
                    alpha = c(0.1,0.2,0.3,0.4,0.5),
                    dfg1 = bamm_dfg_bg, dfg2 = bamm_dfg) %>%
  as.data.frame()
score_dist_is$alpha <- as.factor(score_dist_is$alpha)
score_dist_is$method <- "IS"
```

```r
library(ggplot2)

plot_df <- rbind(score_dist_saddle[,c("x","p","method")],
                 score_dist_is[,c("x","p","method")])

ggplot(plot_df, aes(x = x, y = p, colour = method)) +
  geom_line() +  scale_y_log10() + theme_bw() +
  geom_ribbon(data = score_dist_is, aes(ymax = high, ymin = low, fill = alpha, group = alpha),
            alpha = 0.3)
```

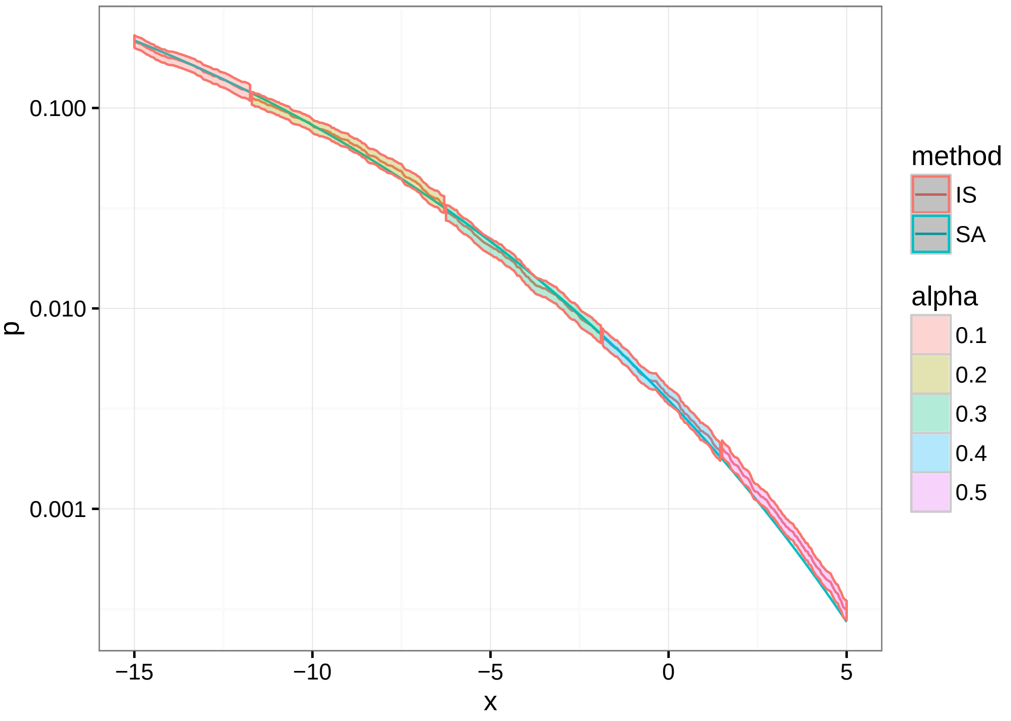

By using different alphas for different parts of the tail. The confidence intervals around the estimates has uniform width on log scale, corresponding to uniform relative error.

xii Example 4: Phylogenetic trees

We used a fixed phylogenetic tree in the example on evolutionary conservation. The tree given in Newick format is:

```
tree_newick <- "(((A:1,B:1):3,((C:1,D:1):2,((E:1,F:1):1,G:2):1):1):1,(((H:1,I:1):1,J:2):1,K:3):2):1;"
```

We scale all branches by a factor of 0.1.

We use the Jukes-Cantor substitution model, but any substitution model can be used. The transition matrix given branch length, t, is given by:

$$P_{ij}(t) = \begin{cases} \frac{1}{4} + \frac{3}{4}e^{-4t} & i = j \\ \frac{1}{4} - \frac{1}{4}e^{-4t} & i \neq j \end{cases}$$

This matrix can be easily calculated for any substitution rate model using matrix exponentiation.

The expected number of substitutions along a branch of length, t, given the endpoints is given by:

$$\mathbb{E}[\, N(t) \mid X(0) = i, X(t) = j] = \begin{cases} \frac{-3te^{-4t}/4+3t/4}{1/4+3e^{-4t}/4} & i = j \\ \frac{te^{-4t}/4+3t/4}{1/4-e^{-4t}/4} & i \neq j \end{cases},$$

where $N(t)$ is the number of substitutions in $[0; t]$. This matrix can be found for other substitution rate models using the so-called Van-Loans method [2].

xiii Phylogenetic tree Vignette

```
library(dgRaph)
library(ggplot2)
library(dplyr)
```

In the following phylogenetics example we will assume the simple Jukes-Cantor model. The state-transition matrix and the expected number of substitions as a function of time, t, can be calculated easily. Using Van Loan's method this can be done for any transition rate matrix.

```
JC_trns <- function(t){
  matrix(1/4 - exp(-4*t)/4, 4,4) + diag(exp(-4*t), 4)
}

JC_subs <- function(t){
  ret <- matrix((t/4*exp(-4*t)+t*3/4)/(1./4-exp(-4*t)/4), 4, 4)
  ret <- ret + diag(-ret[1,1]+(-t*3/4*exp(-4*t) + t*3/4)/(1./4+3*exp(-4*t)/4) , 4 )
}
```

We then build phylogenetic tree with nine leaves.

```
varDim <- rep(4,20)
t <- 0.2
facPot <- list(JC_trns(1*t), JC_trns(2*t), JC_trns(3*t))
facNbs <- list(c(1,12),c(2,12),c(3,13),c(4,13),c(5,14),c(6,14),c(8,15),c(9,15), #1
               c(14,16),c(15,17),c(16,18),c(17,19),c(18,20),  #1
               c(10,17),c(13,18),c(7,16), #2
               c(11,19),c(12,20),c(19,20)) #4

potMap <- c(rep(1,13),2,2,2,3,3,3)
phylo_tree <- dfg(varDim, facPot, facNbs, potMap)
plot(phylo_tree, layout = layout.reingold.tilford)
```

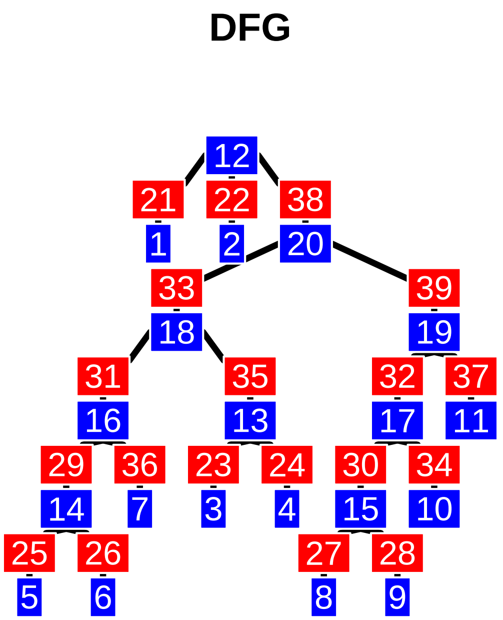

Next we do importance sampling of the expected number of substitions given the leaves are observed. As a we are interested in a surprisingly *small* number of substitions we let the score be the negative number of expected substitutions.

```
facScores <- list(-JC_subs(1*t),
                 -JC_subs(2*t),
                 -JC_subs(3*t))

set.seed(1)
tail_df <- tailIS(x = seq(-19, -12, 0.1), alpha = c(0,1.5), n = 1000,
                  dfg1 = phylo_tree,
                  facScores = facScores,
                  observed = c(rep(T,11), rep(F,9)))

tail_df_rev <- tail_df %>% mutate(x = -x) %>% mutate(alpha = as.factor(alpha))

ggplot(tail_df_rev, aes(x = x, y = p, ymin = low, ymax = high, fill = alpha, group = alpha)) +
  geom_line() +
  theme_bw() + scale_y_log10() +
  geom_ribbon(aes(colour = NULL), alpha = 0.3) +
  scale_x_reverse() + ylab("P(X < x)") + xlab("s")
```

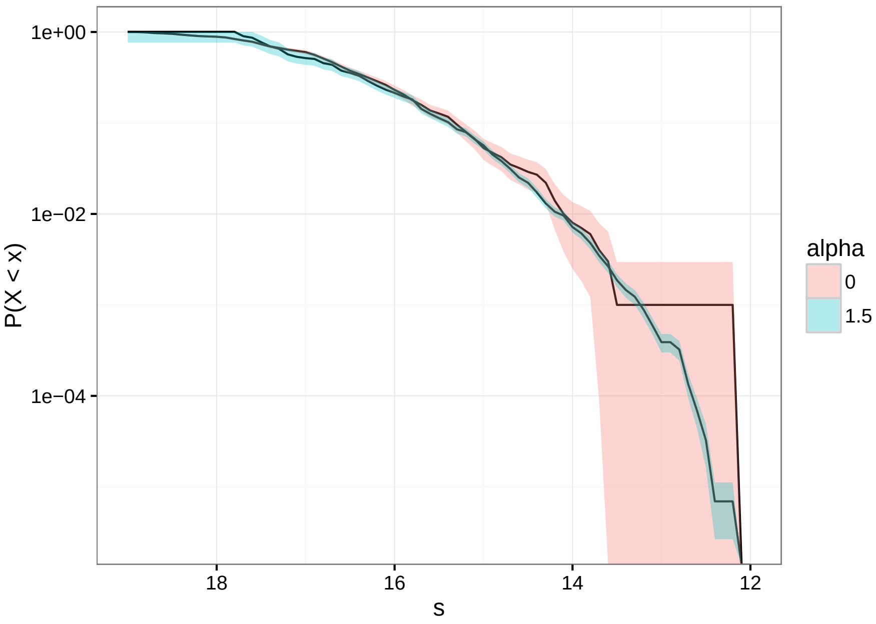

Again notice how the confidence intervals get smaller in the tail using an alpha different from 0.

xiv Asymptotic Results

In this section we state an asymptotic result that can be applied in the case of the Poisson-Binomial and the PWM model. We consider a sequence of variables taking values on $\mathbb{Z}$. This is automatically the case for the Poisson-Binomial model. For the PWM model we can discretize our scores and scale them to meet this requirement.

Consider a sequence of random variables $X_1, X_2, \ldots X_n$, taking values in $\mathbb{Z}$. Define $S_n = \sum_{i=1}^n X_i$. Let $X_i \sim \sum_{j=1}^k p_j^{(i)} \delta(s_j^{(i)})$, where $\delta(\cdot)$ is the dirac measure, $s_j^{(i)} \in \mathbb{Z}$ and $\sum_{j=1}^k p_j^{(i)} = 1$. The moment generating function is given by

$$\varphi_{X_i}(\theta) = \sum_{j=1}^k p_j^{(i)} \exp(\theta s_j^{(i)}).$$

Define the exponentially tilted variable as

$$X_i^\theta \sim \frac{1}{\varphi_{X_i}(\theta)} \sum_{j=1}^k p_j^{(i)} \exp(\theta s_j^{(i)}) \delta(s_j^{(i)}). \tag{S11}$$

Similarly define the sum of the tilted variables, $S_n^\theta = \sum_{i=1}^n X_i^\theta$. Finally let $\sigma_n(\theta) = \left(\frac{1}{n}\mathbb{V}S_n^\theta\right)^{1/2}$.

In this setting we can reformulate Theorem 6.5.4 of [4].

Lemma 1. *Let Θ_0 be a subset of $\mathbb{R}$. Assume there exist constants $a_1 > 0$, $a_2 > 0$ and a set $B_0 \subseteq \mathbb{N}$ such that, for $\theta \in \Theta_0$:*

(i) $\frac{1}{n}\sum_{i=1}^n \mathbb{E}_\theta \left|(X_i - \mathbb{E}_\theta X_i)\sigma_n(\theta)^{-1}\right|^6 \leq a_1$.

(ii) For any $c > 0$ there exist $\rho < 1$ such that for any $i \in B_0$ we have $|\varphi_{X_i}(\theta + ir)/\varphi_{X_i}(\theta)| \leq \rho$ for $c \leq |r| \leq \pi$.

(iii) $\liminf_{n\to\infty} \frac{1}{n} |\{i \in B_0 \mid i \leq n\}| \geq a_2$.

Then the relative error of the saddlepoint approximation to $P(S_n/n > t)$ will be uniformly $\mathcal{O}(1/n)$ for t such that the saddlepoint $\theta(t) \in \Theta_0$.

We will now show useful sufficient conditions under which the assumptions of Lemma 1 are met for the Poisson-Binomial model and the PWM model.

First consider the Poisson-Binomial distribution. Let $p_1, \ldots, p_n$, be a set of probabilities and $X_1, \ldots, X_n$, be independent with $X_i \sim \text{Bernoulli}(p_i)$. In the formulation above we have $k = 2$, $s_1^{(i)} = 1$, $s_2^{(i)} = 0$, $p_1^{(i)} = p_i$ and $p_2^{(i)} = 1 - p_i$.

Proposition 1. *Let Θ_0 be a compact subset of $\mathbb{R}$ and assume there exists $\epsilon > 0$ and $a > 0$ such that $\lim_{n\to\infty} \frac{1}{n} |\{i \in B_0 \mid i \leq n\}| \geq a$ where $B_0 = \{i \mid p_i \in [\epsilon; 1-\epsilon]\}$. Then the assumptions of Lemma 1 are met for the Poisson-Binomial model.*

Proof. The variance for a generic X^θ is

$$\frac{p(1-p)\exp(\theta)}{(1 - p + p\exp(\theta))^2}.$$

This is a continuous function of p and θ and has a lower limit $c_1 > 0$ for (p, θ) in the compact set $[\epsilon; 1-\epsilon] \times \Theta_0$. This gives $ac_1 \leq \sigma_n(\theta)^2 \leq 1/4$. Since also $|(X_i - E_\theta X_i)^6| \leq 1$ we obtain (i).

Similarly, for a generic X

$$|\varphi_X(\theta + ir)/\varphi_X(\theta)| = |1 - p + pe^{\theta + ir}|/(1 - p + e^\theta)$$

is a continuous function of p, θ and r that is strictly less than 1 for $p \in (0, 1)$, $0 < |r| \leq \pi$ and $\theta \in R$. The maximum ρ over $(p, \theta, |r|) \in [\epsilon, 1-\epsilon] \times \Theta_0 \times [c, \pi]$ is therefore less than 1. This gives (ii), and (iii) is fulfilled by assumption. □

Next we consider the PWM model. This is just the initial setup with $k = 4$.

Proposition 2. *Let* Θ_0 *be a compact subset of* $\mathbb{R}$*. Assume there exists* $\epsilon > 0$ *and* $a_1 > 0, a_2 > 0$ *such that* $|s_j^{(i)}| \leq a_1 \,\forall i, j$ *and* $\lim_{n\to\infty} \frac{1}{n} |\{i \in B_0 \mid i \leq n\}| \geq a_2$ *where* $B_0 = \{i \mid X_i \text{ has minimal lattice } \mathbb{Z}, \min_j p_j^{(i)} \geq \epsilon\}$*. Then the assumptions of Lemma 1 are met.*

Proof. That the minimal lattice of X_i is $\mathbb{Z}$ means that there does not exist $a, b \in \mathbb{Z}$ with $b \geq 2$ such that $s_j^{(i)} \in a + b\mathbb{Z}$, $\forall j$. The proof uses the same ideas as in the proof of Proposition 1. For a fixed set of scores $s_j^{(i)}$, $j = 1, \ldots, k$, the variance of $X_\theta^{(i)}$ is a continuous function of $p_j^{(i)}$ and θ, and has a positive lower bound over the compact set with $p_j^{(i)} \geq \epsilon$ and $\theta \in \Theta_0$. Taking the minimum over the possible combinations of $s_j^{(i)}$, $j = 1, \ldots, k$ gives a positive limit c_1 for the variance, and therefore $\sigma_n(\theta)^2 \geq a_2 c_1$. Since also $|(X_i - E_\theta X_i)^6| \leq 2^6 a_1^6$ we obtain (i).

Similarly, for a fixed set of scores $s_j^{(i)}$, $j = 1, \ldots, k$, with minimal lattice $\mathbb{Z}$ the characteristic function $\varphi(\theta + ir)/\varphi(\theta)$ is bounded from above by $\rho_s < 1$ for $p_j^{(i)} \geq \epsilon$ and $\theta \in \Theta_0$. Taking the maximum over the possible combinations of $s_j^{(i)}$, $j = 1, \ldots, k$ gives an upper limit $\rho < 1$. Thus, (ii) has been proved and again (iii) is fulfilled by assumption. □

For PWM's a pseudocount is often employed thus the probabilities are bounded away from 0. The minimal lattice of a variable X_i is $\mathbb{Z}$ if and only if $\gcd(s_2^{(i)} - s_1^{(i)}, s_3^{(i)} - s_1^{(i)}, s_4^{(i)} - s_1^{(i)}) = 1$. The probability that three random numbers drawn uniformly and independently from $\{1, \ldots, n\}$ are coprime is approximately $1/\zeta(3) \approx 0.83$ [6]. This suggest that it is not unreasonable to expect a large fraction of X_i's in a PWM to have minimal lattice $\mathbb{Z}$.

Although these results discuss the limiting behaviour of the error, [4] demonstrates that in many cases the saddlepoint approximation is remarkably good even for small n.

xv Analysis of JASPAR motifs

We compare significance estimates from TFMPvalue [8], Saddlepoint Approximation and Importance sampling for all JASPAR Vertebrate motifs ($N = 519$) [5]. Using the approach in section ii, we determine the significance estimates, $\tilde{P}(S > q)$ in a series of quantiles, $q_1, \ldots q_i$. And proceed by calculating the average of the absolute value of the relative differences for each motif. Sections viii and ix contain the precise description of the background model and construction of the score matrix.

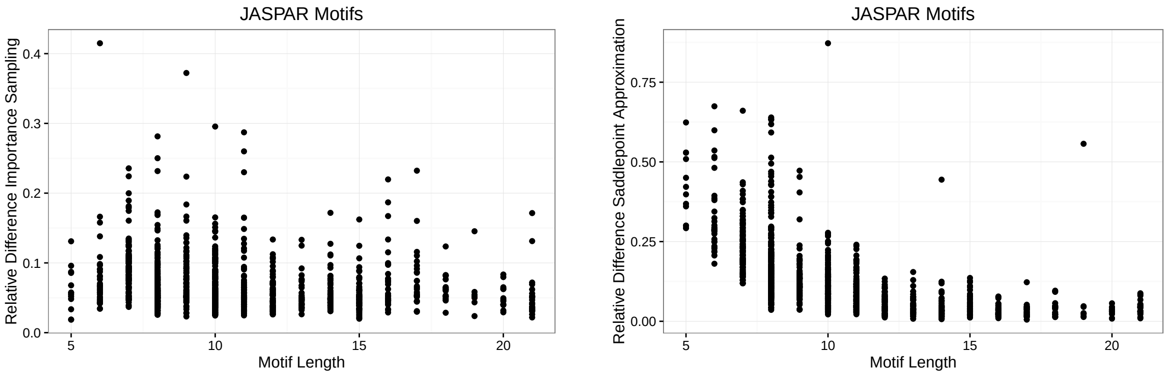

Figure S13: We plotted the relative difference between TFMPvalue significance estimates and Importance Sampling and Saddlepoint Approximation respectively.

References

[1] Richard Arratia, Larry Goldstein, and Louis Gordon. Two moments suffice for Poisson approximations: the Chen-Stein method. *The Annals of Probability*, pages 9–25, 1989.

[2] Asger Hobolth and Jens Ledet Jensen. Summary statistics for endpoint-conditioned continuous-time Markov chains. *Journal of Applied Probability*, pages 911–924, 2011.

[3] Yili Hong. On computing the distribution function for the Poisson binomial distribution. *Computational Statistics & Data Analysis*, 59:41–51, 2013.

[4] Jens Ledet Jensen. *Saddlepoint approximations.* Oxford University Press, 1995.

[5] Anthony Mathelier, Oriol Fornes, David J Arenillas, Chih-yu Chen, Grégoire Denay, Jessica Lee, Wenqiang Shi, Casper Shyr, Ge Tan, Rebecca Worsley-Hunt, et al. JASPAR 2016: A major expansion and update of the open-access database of transcription factor binding profiles. *Nucleic acids research*, 44(D1):D110–D115, 2016.

[6] JE Nymann. On the probability that k positive integers are relatively prime. *Journal of Number Theory*, 4(5):469–473, 1972.

[7] Matthias Siebert and Johannes Söding. Bayesian Markov models consistently outperform PWMs at predicting motifs in nucleotide sequences. *Nucleic acids research*, pages 6055–6069, 2016.

[8] Hélène Touzet and Jean-Stéphane Varré. Efficient and accurate p-value computation for Position Weight Matrices. *Algorithms for Molecular Biology*, 2(1):15, 2007.
